# A vertebrate Vangl2 translational variant required for planar cell polarity

**DOI:** 10.1016/j.jbc.2024.106792

**Published:** 2024-02-24

**Authors:** Alexandra Walton, Virginie Thomé, Diego Revinski, Sylvie Marchetto, Tania M. Puvirajesinghe, Stéphane Audebert, Luc Camoin, Eric Bailly, Laurent Kodjabachian, Jean-Paul Borg

**Affiliations:** 1 Aix Marseille Univ, CNRS, INSERM, Institut Paoli-Calmettes, CRCM, Equipe labellisée Ligue ‘Cell Polarity, Cell Signaling And Cancer’, Marseille, France; 2 Aix Marseille Univ, CNRS, IBDM, Turing Centre for Living Systems, Marseille, France; 3 Aix Marseille Univ, CNRS, INSERM, Institut Paoli-Calmettes, CRCM, Marseille Protéomique, Marseille, France; 4 Institut Universitaire de France (IUF), Paris, France

**Keywords:** vangl2, PCP, *Xenopus*, translation, isoform

## Abstract

First described in the milkweed bug *Oncopeltus fasciatus*, planar cell polarity (PCP) is a developmental process essential for embryogenesis and development of polarized structures in Metazoans. This signaling pathway involves a set of evolutionarily conserved genes encoding transmembrane (Vangl, Frizzled, Celsr) and cytoplasmic (Prickle, Dishevelled) molecules. Vangl2 is of major importance in embryonic development as illustrated by its pivotal role during neural tube closure in human, mouse, *Xenopus*, and zebrafish embryos. Here, we report on the molecular and functional characterization of a Vangl2 isoform, Vangl2-Long, containing an N-terminal extension of about 50 aa, which arises from an alternative near-cognate AUA translation initiation site, lying upstream of the conventional start codon. While missing in Vangl1 paralogs and in all invertebrates, including *Drosophila*, this N-terminal extension is conserved in all vertebrate Vangl2 sequences. We show that Vangl2-Long belongs to a multimeric complex with Vangl1 and Vangl2. Using morpholino oligonucleotides to specifically knockdown Vangl2-Long in *Xenopus*, we found that this isoform is functional and required for embryo extension and neural tube closure. Furthermore, both Vangl2 and Vangl2-Long must be correctly expressed for the polarized distribution of the PCP molecules Pk2 and Dvl1 and for centriole rotational polarity in ciliated epidermal cells. Altogether, our study suggests that Vangl2-Long significantly contributes to the pool of Vangl2 molecules present at the plasma membrane to maintain PCP in vertebrate tissues.

Planar cell polarity (PCP) refers to the cell polarization within the plane of the epithelial sheet and is crucial for the regulation of embryonic development and tissue formation (1). Mammalian Vangl2, known as Van Gogh (Vang)/Strabismus (Stbm) in the fruit fly *Drosophila melanogaster*, is a member of the core PCP proteins, which also comprise Celsr, Frizzled, Dishevelled, and Prickle. Originally identified in the fly as a regulator of eye, wing and bristle development (2, 3), Vang/Stbm is highly conserved during evolution. Like Vang/Stbm, the vertebrate counterparts Vangl1 and Vangl2 (4, 5) contain four transmembrane domains with cytoplasmic amino and carboxy termini and two short loops facing the extracellular space (6, 7, 8). In addition, their carboxy-terminal region ends with a conserved PSD95-Dlg-ZO1 binding motif (3, 9). Previous studies have established that the two vertebrate paralogs interact both genetically (10) and physically (11), with VANGL2 being able to homodimerize and to form heterodimers with VANGL1.

Loss-of-function mutations in human *VANGL1* and *VANGL2* genes are associated with neural tube defects (NTDs) (12, 13), highlighting the importance of these genes in developmental processes. Similarly, mouse mutants with a characteristic *Loop-tail* (*Lp*) phenotype are deficient for Vangl2 functions and *Lp/Lp* homozygous mice display the most severe failure in neural tube closure (craniorachischisis) (14). NTDs result from defective convergent-extension (CE) movements, a collective and polarized migration of neural plate cells during gastrulation and neurulation stages. In *Xenopus* and zebrafish, disrupted CE movements during gastrulation and neurulation due to the depletion of Vangl2 are, respectively, associated with a severe reduction in the body length (15, 16) and incomplete neural tube closure (17, 18, 19, 20, 21). In *Xenopus*, Vangl1 and Vangl2 have also been implicated in PCP deployment within the embryonic epidermis (20, 22). In particular, PCP regulators are necessary in epidermal multiciliated cells (MCCs) to coordinate the orientation of hundreds of centrioles/basal bodies and consequently of cilia, so as to ensure directional beating and effective extracellular fluid propulsion (20, 22, 23).

Most eukaryotic translation initiation events require a canonical AUG start codon to occur near the 5′ cap of messenger RNAs. Yet, a number of translation events has also been shown to occur at near-cognate start codons upstream of and in-frame with the canonical AUG start codon, yielding longer translational variants with potentially distinct (24, 25, 26, 27, 28, 29, 30, 31) and/or overlapping biological functions (32, 33). These alternative translation initiation sites are therefore recognized as an additional source of proteome diversity.

Here, we identify an N terminally extended isoform of human VANGL2, termed VANGL2-Long, which arises from the alternative translation initiation at a near-cognate AUA start codon upstream of the coding region of canonical VANGL2. The sequence encoding this extension is strongly conserved among vertebrate genomes. We show that in *Xenopus laevis*, the Vangl2-Long isoform is indeed expressed during embryogenesis, alongside the shorter canonical isoform. Antisense morpholinos, designed to inhibit specifically Vangl2-Long expression, caused typical PCP phenotypes, such as neural tube closure defects and centriole rotational polarity defects in MCCs, and perturbed asymmetric distribution of Pk2 and Dvl1. Our data reveal the existence of an additional functional isoform of Vangl2, which may contribute to establish PCP in vertebrate tissues.

## Results

### The cell polarity protein VANGL2 is expressed as two different N-terminal isoforms

**Figure 1:**
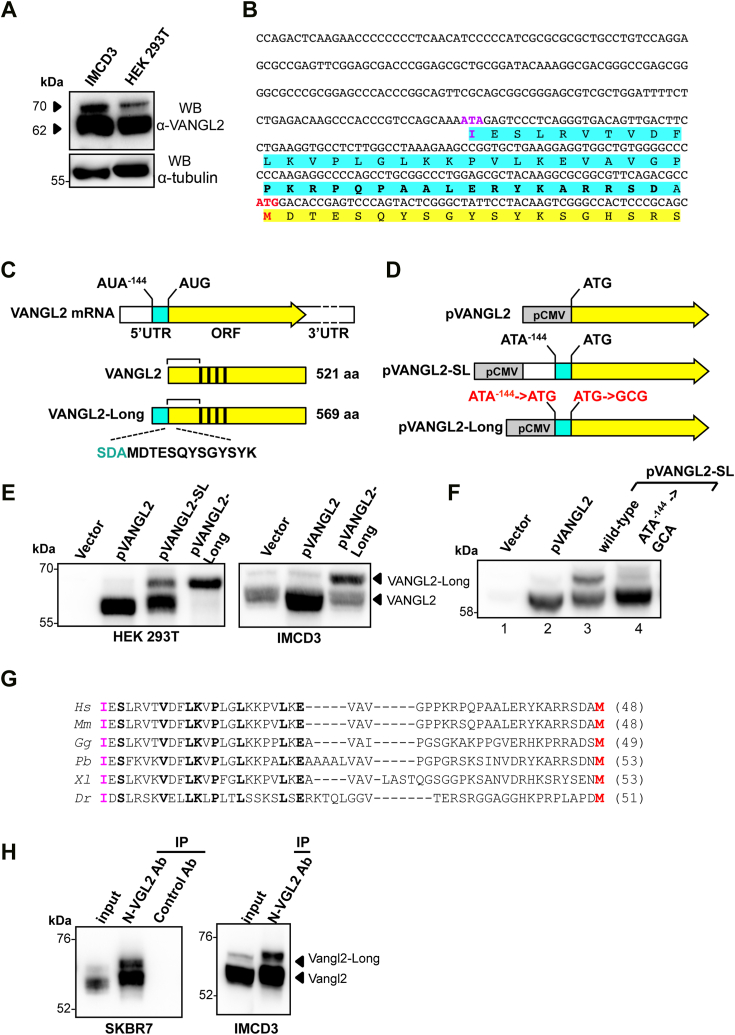
**Identification of VANGL2-Long, a highly conserved N terminally extended VANGL2 isoform, in human and mouse cells.***A*, Western blot analysis of murine epithelial (IMCD3) and human embryonic kidney (HEK 293T) cell extracts using VANGL2-specific mAb 36E3 (*top panel*). *Arrows* point to the major 62 Kd VANGL2 protein and to a less intense and slower migrating band of 70 Kd both recognized by mAb 36E3. Immunoblot with an anti-alpha tubulin (*bottom panel*) was used as a loading control. *B*, DNA sequence of the 5′ region of human VANGL2 cDNA encompassing 342 bp of the 5′-UTR region immediately upstream to the conventional ATG start site (highlighted in *red*) and the first 57 bp of ATG-initiated VANGL2 ORF. A potential ATA alternative initiation site in position −144 (ATA^-144^) relative to the canonical VANGL2 ATG^1^ start site is highlighted in *magenta*. Amino acid sequences of VANGL2 and the N-terminal extension of VANGL2-Long are highlighted in *yellow* and *cyan*, respectively. The sequence of the peptide used to generate the N-VGL2 pAb rabbit antibodies is indicated in *bold letters*. *C*, diagram representations of the human VANGL2 mRNA (*top*) with its canonical (AUG) and near-cognate (ATA^-144^) initiation sites and the two VANGL2 (*middle*) and VANGL2-Long (*bottom*) encoded isoforms. Met-initiated VANGL2 and the ATA-initiated N-terminal extension are drawn in *yellow* and *cyan*, respectively. *Black rectangles* represent the four transmembrane domains present in both isoforms and numbers on the right give their respective length. The sequence of a peptide identified by mass spectrometry and encompassing the N terminus of VANGL2 (*black*) preceded by three residues of the 5′-UTR region (*cyan*) is shown below the VANGL2-Long diagram. *Brackets* above the two schematized isoforms indicate a common region recognized by mAb 36E3. *D*, schemes of pVANGL2 (*top*), pVANGL2-SL (*middle*), and pVANGL2-Long (*bottom*) vectors. All VANGL2 constructs are under the control of CMV promoter (*gray box*) and VANGL2 isoform representation uses the same color code as in (*C*). Note that, as indicated in *red* in pVangl2-Long, ATA^-144^ and the canonical ATG codon have been mutated to, respectively, ATG and GCG in order to enhance VANGL2-Long biosynthesis while abrogating VANGL2 expression by this construct. *E*, HEK 293T (*left panel*) and IMCD3 (*right panel*) cells were probed by immunoblotting with anti-VANGL2 mAb 36E3 upon transfection with the indicated plasmids. Note that the absence of endogenous VANGL2 signals in HEK 293T cells transfected with an empty plasmid (vector) is due to the short exposure time used to detect the overexpressed VANGL2 isoforms. *Arrows* point to VANGL2 and VANGL2-Long isoforms that are overexpressed by the relevant pVANGL2 and pVANGL2-Long constructs. Note that pVANGL2 SL promotes the synthesis of both VANGL2 and VANGL2-Long simultaneously (*left panel*). *F*, VANGL2-L expression requires a near-cognate AUA alternative initiation site in position −144. HEK293T cells transfected with either an empty plasmid (vector), p VANGL2, or pVANGL2-SL plasmids in which the predicted alternative AUA-144 start codon was either left intact (AUA-144) or mutated to GCA (GCA-144) were processed for Western blot analysis with mAb36E3 to detect all VANGL2 isoforms. *G*, amino acid sequence alignment of predicted N-terminal extensions from six vertebrate species, including human (*Hs*), mouse (*Ms*), chicken (*Gg*), python (*Pb*), African clawed frog (*Xl*), and zebrafish (*Dr*). Strictly conserved residues are in *bold letters*. AUA-encoded isoleucine residues that initiate the translation of all the extensions shown here are highlighted in *magenta*, whereas the AUG-encoded methionine residue of Vangl2 is in *red*. *H*, proteins extracted from SKBR7 (*left panel*) and IMCD3 (*right panel*) cells were immunoprecipitated with the VANGL2-Long isoform-specific N-Vgl2 pAbs and immunopurified proteins were immunoblotted with mAb 36E3. Control Ab is an isotypic control rabbit antibody. pAb, polyclonal antibody.

In a previous study, we undertook to generate a collection of mAbs directed against the N-terminal sequence of the human VANGL2 protein (11). Biochemical characterization of a new clone, mAb 36E3, by Western blot (WB) revealed that, in addition to the main 62 kD band corresponding to the 521 aa–long VANGL2 polypeptide, this antibody recognizes a second antigen with an apparent molecular weight of ≈70 kD in both human HEK 293T and murine IMCD3 cell extracts (Fig. 1*A*). This 70 kD band is only observed in VANGL2-positive cells and is always of lower intensity than the major 62 kD VANGL2 signal in all cell lines tested (IMCD3, HEK 293T, SKBR7) (Fig. 1, *A* and *H*). Of note, mAb 2G4, whose specificity against VANGL2 has been demonstrated in a previous work, produces an identical pattern by immunoblotting, with the detection of a major 62 kD band and a weaker signal at ≈70 kD in different murine tissues (11). Because CRISPR-Cas9 editing of *Vangl2* abrogated the expression of both antigens (Fig. S1*A*), we undertook to determine whether the additional 70 kDa signal could represent a previously unrecognized Vangl2 isoform. While protein isoforms can arise from many different molecular events including posttranslational modifications, alternative splicing, or alternative translation initiation, we focused our attention on the latter scenario for two reasons. First, in the course of a previous mass spectrometry (MS) analysis in SKBR7 cells (11), we could establish the existence of a peptide that in addition to the N terminus of VANGL2, contained an upstream SDA sequence (Fig. 1*C*), raising the possibility of an N terminally extended VANGL2 isoform. Second, and fully consistent with this hypothesis, an *in silico* study has suggested the presence of a near-cognate translation initiation AUA codon lying 144 nucleotides upstream of the canonical AUG initiation codon of VANGL2 (Fig. 1*B*) (34).

To ascertain that the 5′UTR region of *VANGL2* mRNA can direct the translation of the predicted VANGL2-Long isoform, we designed three expression plasmids (Fig. 1*D*). In the first construct (pVANGL2), VANGL2 ORF initiated by the canonical ATG start codon was cloned downstream of the strong CMV promoter. A second plasmid (pVANGL2-SL) was a 5′ extended version of pVANGL2 in which the 342 bp region immediately upstream of the VANGL2 ORF (Fig. 1*D*) was inserted in frame with the ATG start codon of VANGL2. A third vector (pVANGL2-Long) was specifically generated for the expression of a 569 aa VANGL2-Long polypeptide, using an appropriately designed cDNA as depicted in Figure 1*D*. Transfection experiments in HEK 293T and IMCD3 cells indicated that pVANGL2 and pVANGL2-Long plasmids each drove the synthesis of a single polypeptide of, respectively, 62 and 70 kD, which perfectly comigrated with the endogenous VANGL2 signals detected by mAb 36E3 (Fig. 1*E*). These results indicate that the 70 kD band likely corresponds to VANGL2-Long. Remarkably, in the same transfection assay, pVANGL2-SL simultaneously gave rise to two distinct 62 and 70 kD VANGL2 products with a VANGL2: VANGL2-Long ratio very similar to the ratio observed in mammalian cell extracts (compare left panel of Fig. 1, *A*–*E*). The latter result therefore established the ability of the 5′cap mRNA to initiate the translation of a longer and less abundant VANGL2 isoform, in addition to the major canonical VANGL2 protein. In support of a critical role of the ATA-144 codon, a GCA mutation at this position in pVANGL2-SL abrogated the expression of the 70 kDa antigen without altering the levels of VANGL2 (Fig. 1*F*, compare lanes 3 and 4). Taken together, these data provide strong experimental evidence for the requirement of AUA^144^ in the alternative translation initiation of a novel VANGL2 isoform, which differs from the canonical isoform by the presence of a 48 aa N-terminal extension.

The ability of the *VANGL2* locus to encode a longer isoform from a noncanonical AUA start codon is not limited to human but predicted to exist in all vertebrates for which a Vangl2 sequence is currently available. In contrast, the N-terminal extension coding sequence is found neither in the *Vangl1* mRNA nor in the *Vang/Stbm* coding sequences of more distantly related species, such as *D. melanogaster* or *Caenorhabditis elegans*. Protein sequence comparison between the N-terminal extensions of human (Hs), mouse (Ms), chicken (Gg), python (Pb), African clawed frog (Xl), and zebrafish (Dr) confirmed the high degree of phylogenetic conservation of this motif (Fig. 1*G*). This is best illustrated by the presence of several invariant residues along the extension of all these vertebrate members. The protein sequence of the 48 aa Vangl2-Long extension has, however, no homology with any other sequences available in protein databases.

We next sought to validate the expression of VANGL2-Long in mammalian cell lines, using rabbit polyclonal antibodies (hereafter referred to as N-VGL2 pAb) raised against a peptide present within the N-terminal extension of VANGL2-Long (Fig. 1*B*, aa sequence in bold letters). An immunoprecipitation (IP) assay of protein extracts from IMCD3 cells that were engineered to express either of the two VANGL2 isoforms confirmed the ability of N-VGL2 pAb to recognize VANGL2-Long but not VANGL2 (Fig. S1*B*). Further IP experiments with this antibody allowed us to demonstrate the occurrence of endogenous VANGL2-Long in human (SKBR7) and murine (IMCD3) cells (Fig. 1*H*) as evidenced by the strong enrichment of a 70 kD 36E3-reactive signal in the N-VGL2 pAb IP. Surprisingly, the 62 kD VANGL2 protein was also massively detected in the material immunoprecipitated by N-VGL2 pAb and not with a control antibody (Fig. 1*H*, left panel). Since N-VGL2 pAb does not recognize VANGL2 alone (Fig. S1*B*), the latter observation led us to conclude to the existence of a physical interaction between VANGL2 and VANGL2-Long. Taken together, the biochemical data described above clearly identify the 70 kD protein as a naturally occurring VANGL2-Long isoform in human and murine cells and establish its ability to form an endogenous complex with VANGL2.

### VANGL2-long forms a multimeric complex with VANGL1 and VANGL2

**Figure 2:**
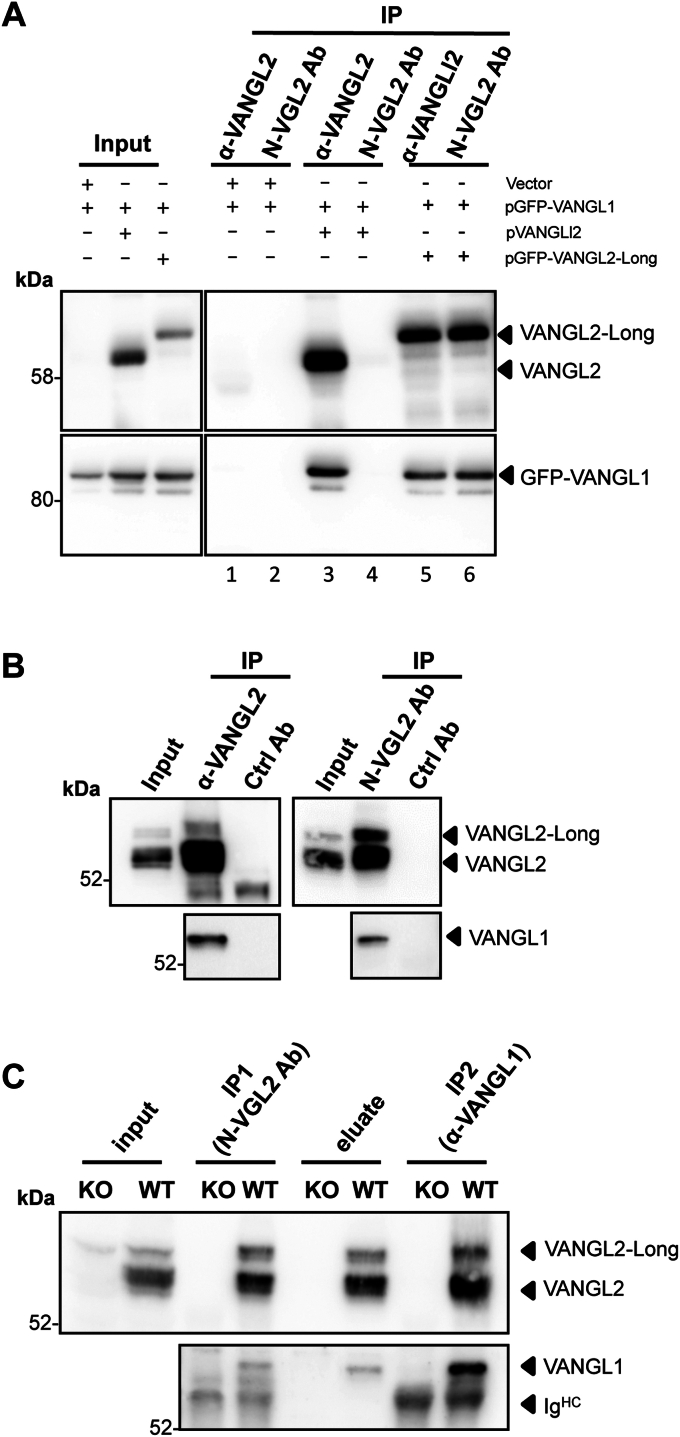
**VANGL2 and VANGL2-Long form a tripartite complex with Vangl1 *in vivo*.***A*, immunoprecipitation of lysates from HEK 293T cells transfected with GFP-VANGL1 and a control plasmid or GFP-VANGL1 in combination with pVANGL2 or pVANGL2-Long, using mAb 36E3 or N-VGL2 pAbs, as indicated. The presence of GFP-VANGL1 and VANGL2 in the immunoprecipitated samples was determined by Western blotting using, respectively, a GFP-specific antibody (*bottom panels*) and mAb 36E3 (*top panels*). *Arrows* point to the VANGL2 and VANGL2-Long signals detected by mAb 36E3. *B*, SKBR7 cell extracts were immunoprecipitated with either mAb 36E3 (*left panels*) or N-VGL2 pAb (*right panels*), followed by a Western blot analysis of VANGL1 (*bottom panels*) and VANGL2 isoforms (*top panels*) using, respectively, mAb 19D5 and mAb 36E3 on two different membranes. Isotopic rat and rabbit antibodies were used as negative controls for each IP experiment (Ctrl Ab). *C*, lysates from WT and Crispr-Cas9 KO-*VANGL2* (KO) HEK 293T cells were first immunoprecipitated with N-VGL2 pAb (IP1, N-VGL2 Ab). Bound proteins were subsequently eluted with the relevant immunogenic peptide (eluate) before being subjected to a second round of IP with VANGL1-specific mAb 19D5 (IP2, α-VANGL1). The different fractions were examined by immunoblotting for their VANGL1 (*lower panel*) and VANGL2 (*upper panel*) contents using, respectively, mAb 19D5 and mAb 36E3. *Arrows* in the *upper panel* indicate the two endogenous VANGL2 and VANGL2-Long isoforms, while those in the *lower panel* point to Vangl1 and immunoglobulin heavy chains (Ig^HC^). pAb, polyclonal antibody.

The two paralogs VANGL1 and VANGL2 are known to interact both genetically (10) and physically (11). To assess whether VANGL2-Long can similarly hetero-oligomerize with VANGL1, HEK 293T cells expressing GFP-VANGL1 alone or in combination with VANGL2 or VANGL2-Long were subjected to IP with mAb 36E3 or N-VGL2 pAb (Fig. 2*A*). This experiment first confirmed previous reports showing the physical interaction between VANGL1 and VANGL2 (Fig. 2*A*, lane 3). It also showed that GFP-VANGL1 copurifies with VANGL2-Long regardless of the antibody used for the IP (Fig. 2*A*, lanes 5 and 6). This interaction was independent of the presence of endogenous VANGL2. Thus, we conclude that VANGL2-Long, like VANGL2, is able to interact with VANGL1.

To determine if VANGL2-Long also interacts with VANGL1 when these proteins are endogenously expressed, we took advantage of our recently developed VANGL1-specific mAbs previously validated by WB (Fig. S2, *A* and *C*) and IP (Fig. S2, *B* and *C*). We used our VANGL1-specific mAb 19D5 to monitor the presence of VANGL1 among the proteins immunoprecipitated from SKBR7 cell lysates with either mAb 36E3 or N-VGL2 pAbs. VANGL1 was readily coimmunoprecipitated by mAb 36E3 (Fig. 2*B*, left panel) and N-VGL2 pAb (Fig. 2*B*, right panel), demonstrating the capacity of VANGL1 to form a protein complex with both VANGL2 and VANGL2-Long isoforms.

The biochemical data presented above point to a unique property of the three VANGL1/2/2-Long proteins to physically interact with one another in human cell lines. Yet, the critical question remained as to whether these proteins could assemble into a single macromolecular complex. We addressed this question by performing a sequential IP assay in which the VANGL2-Long isoform and its associated proteins were first immunopurified from HEK 293T cells with N-VGL2 pAb (Fig. 2*C*, IP1 N-Vgl2 Ab). A *VANGL2*-KO HEK 293T cell line was used as a negative control for this experiment. In a second step, proteins retained by N-VGL2 pAb antibody were eluted with the peptide used for its production. Finally, the eluate fraction was subjected to a second round of IP using our VANGL1-specific mAb 19D5 (Fig. 2*C*, IP2 α-VANGL1). WB analysis was then conducted to monitor VANGL1 and VANGL2 contents of the different fractions. As expected from the findings illustrated in Figures 1*H* and 2*B*, IP with N-VGL2 pAb resulted in a substantial enrichment of VANGL2-Long but also VANGL2 and VANGL1 (Fig. 2*C*, IP1). Importantly, all these proteins were successfully eluted from the beads as judged by the identical VANGL1 and VANGL2/2-Long profiles exhibited by the N-VGL2 pAb IP and eluate fractions (compare fourth and sixth lanes of Fig. 2*C*). The finding that large amounts of both VANGL2 and VANGL2-Long isoforms were still present after VANGL1 IP, unambiguously demonstrated the ability of the three proteins to assemble into a single macromolecular complex (Fig. 2*C*, IP2). Notably, using IMCD3 cells, we found that the Vangl2:Vangl2-Long ratio in this complex range from 3:1 to 4:1, whereas the ratio within cell extracts is 8.5:1 (Fig. S3, *A* and *B*) suggesting that about 50% of Vangl2 is not in complex with Vangl2-Long. We performed a serial IP protocol in IMCD3 cell extracts to test this hypothesis and noticed that Vangl2, unlike Vangl2-Long, could be depleted only partially (about 50%) even after two successive rounds of IP with N-VGL2 pAb (Fig. S3*C*, lanes 1–5). We reasoned that the Vangl2 fraction not copurifying with Vangl2-Long might be in a distinct complex with Vangl1 only. This hypothesis was experimentally tested by subjecting Vangl2-Long depleted lysates (Fig. 2*D*, lane 5) to a novel IP with a Vangl1-specific antibody (Fig. 2*D*, lane 6). Vangl2 was highly enriched in this Vangl1 IP and strongly depleted in the corresponding unbound fraction (Fig. 2*D*, lane 7). These data are thus consistent with the existence of at least two types of VANGL oligomers in human and mouse cells: a tripartite complex that includes VANGL1, VANGL2, and VANGL2-Long and a simpler hetero-oligomer consisting of VANGL1 and VANGL2.

### Vangl2-Long is expressed and polarized in *Xenopus* embryonic cells

In the absence of a robust experimental PCP model in mammalian cultured cells, we decided to turn to the early embryonic development in *Xenopus* to address the functional relevance of Vangl2-Long. Using *Xenopus* xVangl1, xVangl2, and xVangl2-Long ORFs fused to GFP, we found that mAb 36E3 was able to detect both isoforms of xVangl2 but not xVangl1 by WB (Fig. S4*A*). We took advantage of the immunoreactivity of mAb 36E3 to assess the existence of a *Xenopus* Vangl2-Long isoform. WB analysis of kidney epithelial *Xenopus* A6 cells gave a Vangl2 pattern that provided a first piece of evidence, as mAb 36E3 could detect, like in SKBR7 cells, two distinct bands of 62 kD and a 70 kD (Fig. S4*B*). Next, we used MS to analyze proteins immunoprecipitated from gastrula stage *Xenopus* embryos by mAb 36E3 and recovered several peptides corresponding to proteins encoded by the *vangl2-L* and/or *vangl2-S* homologs (Fig. S4*C* and Table S1). Two peptides were of particular interest, as one started at the expected Met-initiated N terminus of Vangl2, while the other contained an additional stretch of four N-terminal amino acid, the sequence of which perfectly matched the last four residues of the predicted N-terminal extension of Vangl2-Long (Fig. S4*D* and Table S1).

The biochemical data reported above together with our bioinformatic analysis of the 5′-cap region of the *xVangl2* loci strongly argue in favor of an alternatively translated xVangl2-Long isoform in this species. It should be noted here that our MS analysis of the polypeptides immunoprecipitated by mAb 36E3 also recovered four xVangl1 peptides, further extending the interaction data obtained in mouse and human cells (Table S2) (11).

**Figure 3:**
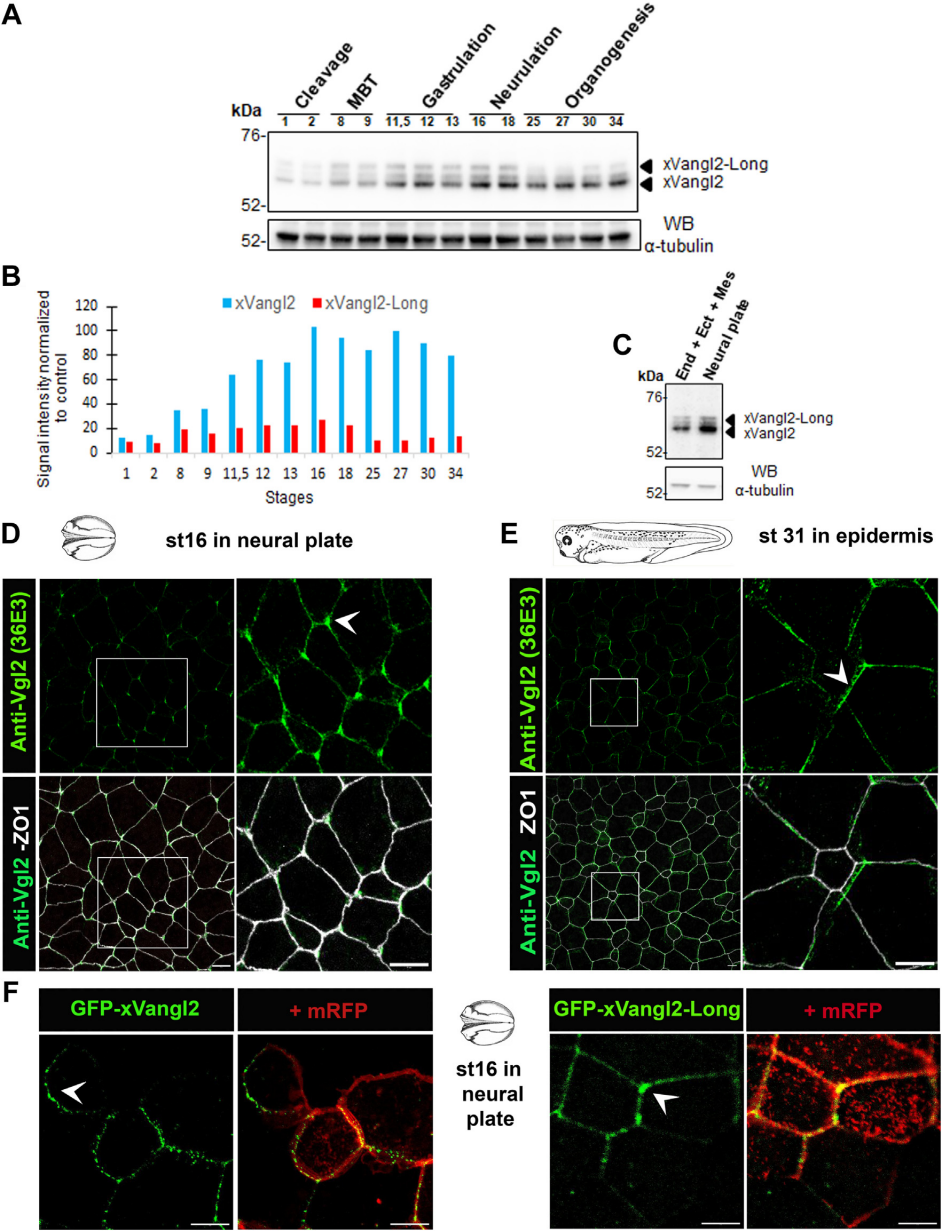
**xVangl2-Long expression during *Xenopus* embryogenesis.***A*, whole *Xenopus* embryos collected at the indicated stages of development were used for protein extraction and Western blot analysis with mAb 36E3 (*upper panel*). Anti-tubulin immunoblotting was used to control protein loading (*lower panel*). The bands corresponding to xVangl2 and xVangl2-Long are indicated by *black arrowheads*. *B*, *graphical representation* of xVangl2 and xVangl2-Long normalized levels corresponding to the blot shown in (*A*). *C*, embryos at stage 14 were dissected to isolate the neural plate from the rest of the embryo, which comprised endodermal, ectodermal, and mesodermal tissues (End + Ect + Mes). Proteins were extracted and probed by Western blotting with mAb 36E3 (*upper panel*) and anti-tubulin (*lower panel*). *D* and *E*, spatial distribution of Vangl2 proteins revealed by immunofluorescence (IF) with mAb 36E3 in neural plate cells at stage 16 (*D*) and in epidermis at stage 31 (*E*). ZO1 IF was used to delineate cell junctions. *White arrowheads* point to polarized Vangl2 enrichment in the plasma membrane, which is anterior in neural plate cells and posterior in epidermal cells. *F*, GFP-xVangl2 and GFP-xVangl2-Long synthetic mRNAs were injected in dorsal blastomeres at 4-cell stage to reveal the spatial distribution of either Vangl2 isoform in neural plate cells of live stage 16 embryos. mRFP mRNA coinjection was used to delineate cell contours. *White arrowheads* point to anterior enrichment of Vangl2 and Vangl2-Long. In all panels, the scale bars represent 10 μm. MBT, midblastula transition.

Next, we monitored the levels of expression of the canonical and extended Vangl2 isoforms, by Western blotting with mAb 36E3, during *Xenopus* embryogenesis (Fig. 3, *A* and *B*). Both isoforms are detectable from the very beginning of embryogenesis at cleavage stages, presumably from maternal origin (stage 1). At midblastula transition (stages 8–9), expression of both xVangl2 and xVangl2-long starts to increase, reaching its highest levels during gastrulation and neurulation. xVangl2 remained highly expressed from gastrulation to organogenesis. In contrast, a significant decline in xVangl2-Long levels occurred from tail bud stage 25. These results indicate that xVangl2 expression and the alternative translation of xVangl2-Long are both subjected to a precise developmentally regulated program (Fig. 3*B*). To gain knowledge about global tissue distribution of xVangl2-Long, we probed extracts from dissected parts of early neurula embryos, by immunoblotting with mAb 36E3 (Fig. 3*C*). xVangl2 and xVangl2-Long were found to be slightly enriched in the neuroectoderm relative to the rest of the embryo.

Next, we used mAb 36E3 in immunofluorescence (IF) assays to monitor the spatial distribution of endogenous xVangl2 proteins in the neural plate at stage 16 and the epidermis at stage 31, where PCP signaling is known to be active (20, 22, 35, 36) (Fig. 3*D*). In the neural plate at stage 16, we observed the presence of xVangl2 at the plasma membrane with a strong accumulation of the signal at the anterior edge of the cells (Fig. 3*D*), consistent with a previous report (35). In the mature epidermis, the xVangl2 signal was also localized at the cell membrane, with a posterior enrichment (Fig. 3*E*), consistent with the previously reported posterior enrichment of GFP-Vangl1 (22). As our antibody could not distinguish the long and conventional xVangl2 isoforms in IF assays, we microinjected synthetic RNAs encoding GFP-xVangl2 or GFP-xVangl2-Long and compared their distribution in neural plate cells at stage 16 (Fig. 3*F*). This experiment confirmed the localization of both isoforms at the plasma membrane, with a clear enrichment at the anterior edge of the cells, as observed for endogenous xVangl2 proteins.

Altogether, our data confirm the existence of the long isoform of Vangl2 in *Xenopus* embryos.

### xVangl2-Long is required for axis elongation and neural tube closure

**Figure 4:**
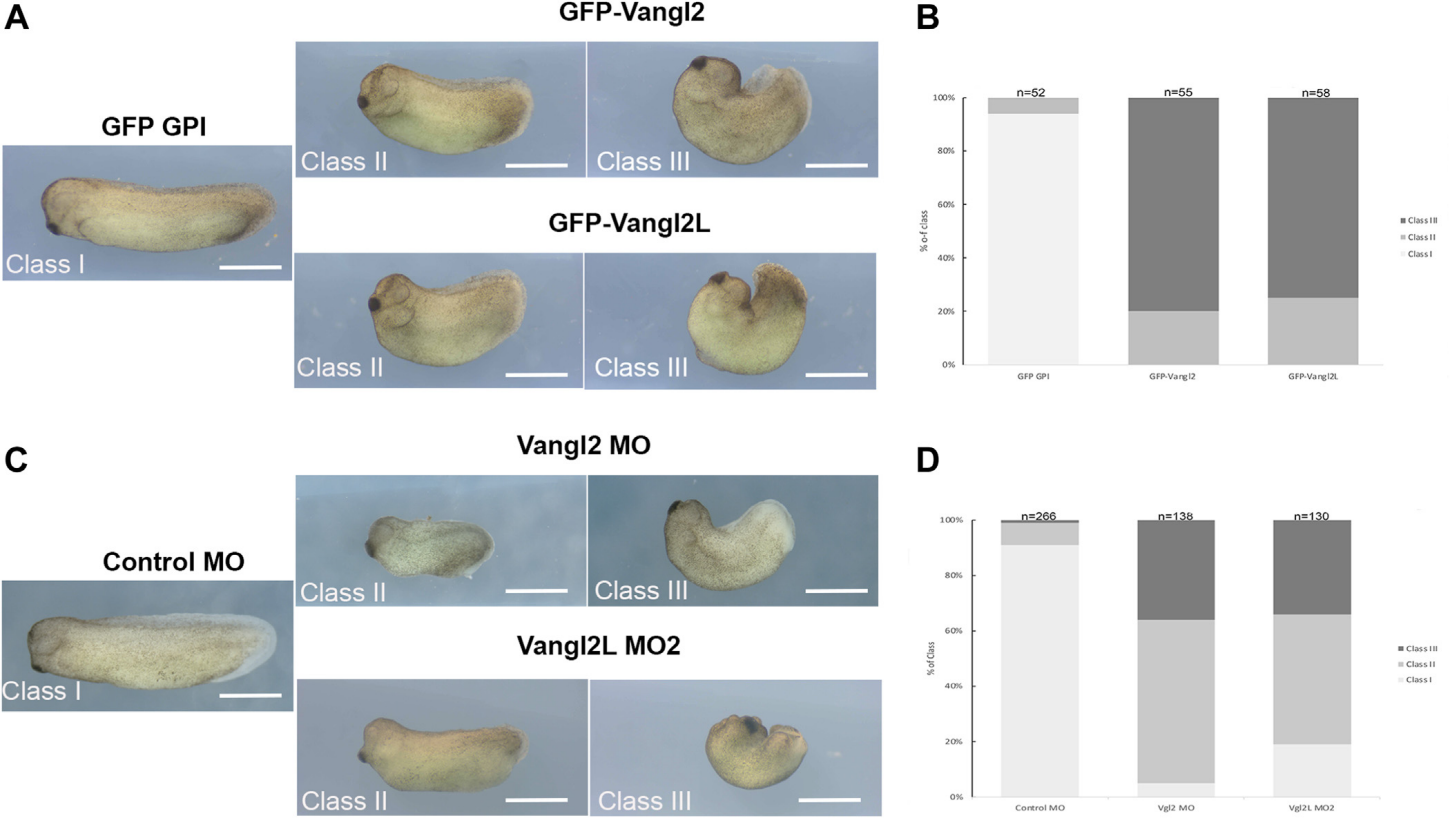
**Vangl2-Long overexpression and knockdown cause strong morphogenetic defects in *Xenopus* embryos.***A*, *Xenopus* embryos were injected with RNA coding for GFP-GPI as control in dorsal blastomeres at 4-cell stage and grown until stage stages 28 to 30. Overexpression of GFP-Vangl2 and GFP-Vangl2-Long caused body axis shortening (class II), or severe bending and defective neural tube closure (class III). In all panels, the scale bars represent 1 mm. *B*, histogram showing the class distribution of embryos injected as indicated. The bars represent the proportions of embryos in each class calculated for five independent experiments. The total number of embryos analyzed for each condition is given above each bar. *C*, 4-cell embryos were injected with control MO or MOs directed against Vangl2 (Vangl2 MO) and Vangl2-Long (Vangl2-Long MO2) in the marginal zone and grown until stage 31. In all panels, the scale bars represent 1 mm. *D*, histogram showing the class distribution of embryos injected as indicated. The bars represent the proportions of embryos in each class calculated for seven independent experiments. The total number of embryos analyzed for each condition is given above each bar. For both overexpression and knockdown assays, the statistical significance of the observed phenotypes was confirmed by a Pearson’s Chi-squared test between class II or class III *versus* class I, with a *p*-value<2.2e16. MO, morpholino oligonucleotide.

As a first test to compare the biological activities of the short and long Vangl2 isoforms, we microinjected synthetic RNAs encoding GFP-xVangl2 or GFP-xVangl2-Long, in dorsal blastomeres of 4-cell embryos, and quantified the consequences on body axis development (Fig. 4). Consistent with previous reports (18, 20, 21), xVangl2 overexpression caused morphological defects ranging from body axis shortening (class II, 20%) to severe bending and incomplete dorsal closure (class III, 80%) (Fig. 4, *A* and *B*). xVangl2-Long overexpression caused a similar range of phenotypes (25% class II, 75% class III) (Fig. 4, *A* and *B*), indicating that convergent extension movement was strongly affected by either Vangl2 isoform.

Next, to evaluate the relative importance of the two endogenous Vangl2 isoforms for embryogenesis, we used morpholino oligonucleotides (MOs), designed to inhibit the translation of the conventional (Vangl2 MO, (20)) or long (Vangl2-Long MO1 and Vangl2-Long MO2) isoforms. First, a survival dose-response curve was established to determine sublethal doses of MOs to be used in subsequent functional assays (Fig. S5*A*). Next, MO specificity was assessed by WB analysis with mAb 36E3 (Fig. S5*B*). While Vangl2 MO reduced the levels of xVangl2 by 50%, it had a similar impact on xVangl2-Long expression. In contrast, the effect of Vangl2-Long MO1 and MO2 was found to be more specific, as judged by their high potency at downregulating xVangl2-Long (about 70% reduction), and their minor impact on the expression of xVangl2 (less than 20% reduction)(Fig. S5*C*). Intriguingly, WB analysis revealed the occurrence of an intermediate band between xVangl2 and xVangl2-Long in protein extracts from whole *Xenopus* embryos (Figs. 3*A* and S5*B*). The intensity of this band was also reduced by about 50% upon injection of either of our three MOs, suggesting that it indeed corresponds to a Vangl2 polypeptide (Fig. S5*C*). The capacity of Vangl2 MO and Vangl2-Long MO2 to inhibit translation was also evidenced by the reduction of mAb 36E3 IF signals in neural plate cells injected with either morpholino (Fig. S5, *D* and *D′*).

As reported in previous works (18, 19, 20, 21), Vangl2 MO severely impaired morphogenesis in *Xenopus*, causing 59% class II and 36% class III embryos (Fig. 4, *C* and *D*). Strikingly, a similar proportion of class II (47%) and class III (34%) embryos was recorded upon Vangl2-Long knockdown by MO1 or MO2 (Figs. 4, *C* and *D*, and S6*A*). As the two MOs against xVangl2-Long caused similar levels of inhibition and morphogenetic defects (Figs. S5, *B* and *C* and S6*A*), only Vangl2-Long MO2 was employed for the rest of the study.

To assess the specificity of our morpholinos toward Vangl2 proteins, we sought to design a rescue assay. Previous studies have revealed the difficulty to achieve rescue for PCP gene knockdown, as depletion and overexpression typically cause the same defects (18, 20, 22). One interesting exception, however, concerns the rescue of ciliogenesis in Vangl2 morphant MCCs, reported by Mitchell *et al.* (2009). Immunofluorescent staining with mAb 36E3 confirmed that Vangl2 proteins are enriched in MCCs, at the time when ciliogenesis is engaged (Fig. S6*B*). Both Vangl2 MO and Vangl2-Long MO2 dramatically reduced the proportion of normally ciliated cells. This phenotype was efficiently corrected by coinjection of *vangl2* and *vangl2-Long* mRNAs, which lacked sequences targeted by the MOs, respectively (Fig. S6, *C* and *C′*). Interestingly, efficient rescue was also achieved when *vangl2* was injected in Vangl2-Long morphants and vice versa (Fig. S6, *C* and *C′*).

Altogether, these results strongly suggest that xVangl2-Long contributes an important function during *Xenopus* embryonic development, presumably through its implication in PCP.

### xVangl2-Long is required for asymmetric distribution of PCP molecules

**Figure 5:**
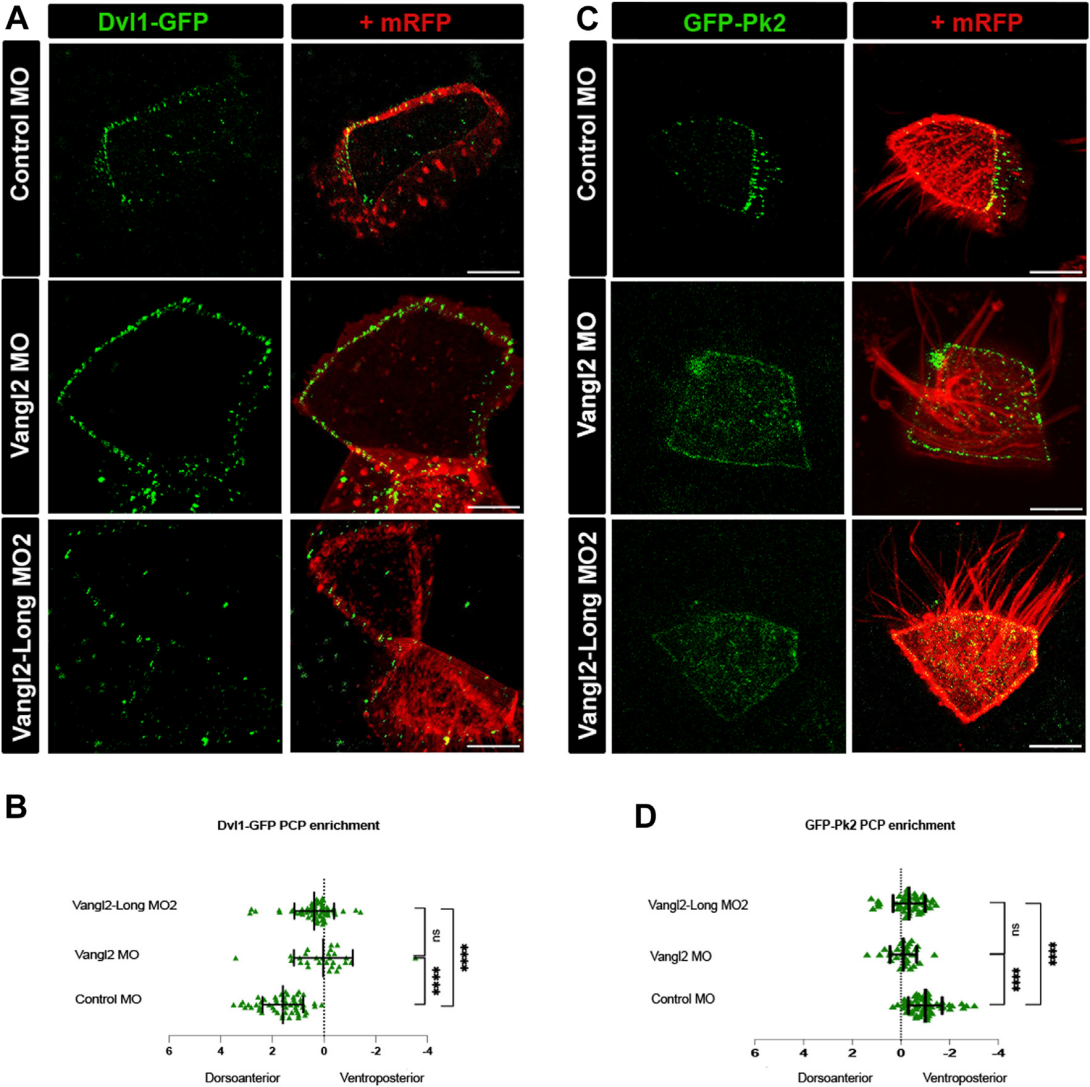
**Vangl2 and Vangl2-Long knockdown disrupts core PCP protein asymmetric distribution.***A*, synthetic mRNAs encoding Dvl1-GFP and mRFP were injected at the 16-cell stage in one ventral animal blastomere, with control, Vangl2 or Vangl2-Long morpholinos. Fluorescent protein localization in epidermal cells was recorded in live embryos at stage 31. In all panels, the scale bars represent 10 μm. *B*, *graph* displaying the ratio of Dvl1-GFP signal intensity between dorsoanterior and ventroposterior domains of the cell membrane. Note that the dorsoanterior enrichment of Dvl1-GFP in control morphant cells (n = 59) is reduced in Vangl2 (n = 28) and Vangl2-Long (n = 65) morphant cells. *C*, synthetic mRNAs encoding GFP-Pk2 and mRFP were injected at the 16-cell stage in one ventral animal blastomere, with control, Vangl2 or Vangl2-Long morpholinos. Fluorescent protein localization in epidermal cells was recorded in live embryos at stage 31. In all panels, the scale bars represent 10 μm. *D*, *graph* displaying the ratio of GFP-Pk2 signal intensity between dorsoanterior and ventroposterior domains of the cell membrane. Note that the ventroposterior enrichment of GFP-Pk2 in control morphant cells (n = 23) is reduced in Vangl2 (n = 28) and Vangl2-Long (n = 48) morphant cells. Statistical analyses were done using GraphPad Prism software, with Student’s *t* tests to evaluate significance. PCP, planar cell polarity.

To assess more directly whether xVangl2-Long is necessary for the proper deployment of PCP, we quantified the polarized distribution of the core PCP molecules Dvl1 and Pk2, in stage 31 epidermal cells of control and morphant embryos. To this end, we microinjected synthetic RNAs coding for Dvl1-GFP or GFP-Pk2 (22). As previously reported (22), in control cells, whether ciliated or not, Dvl1-GFP was significantly enriched in the dorsoanterior quadrant of the plasma membrane (Fig. 5*A*, upper panels and Fig. 5*B*), whereas GFP-Pk2 was enriched in the ventroposterior quadrant (Fig. 5*C*, upper panels and Fig. 5*D*). In cells injected with Vangl2 MO, asymmetric distribution was lost for both Dvl1-GFP (Fig. 5*A*, middle panels and Fig. 5*B*) and Pk2-GFP (Fig. 5*C*, middle panels and Fig. 5*D*). Importantly, Vangl2-Long MO2 caused comparable, albeit slightly weaker, loss of dorsoanterior enrichment of Dvl1-GFP (Fig. 5*A*, lower panels and Fig. 5*B*), and ventroposterior enrichment of Pk2-GFP (Fig. 5*C*, lower panels and Fig. 5*D*).

We conclude that xVangl2-Long is necessary for the polarized distribution of core PCP molecules in mature *Xenopus* epidermal cells.

### xVangl2-Long is required for centriole rotational polarity in MCCs

**Figure 6:**
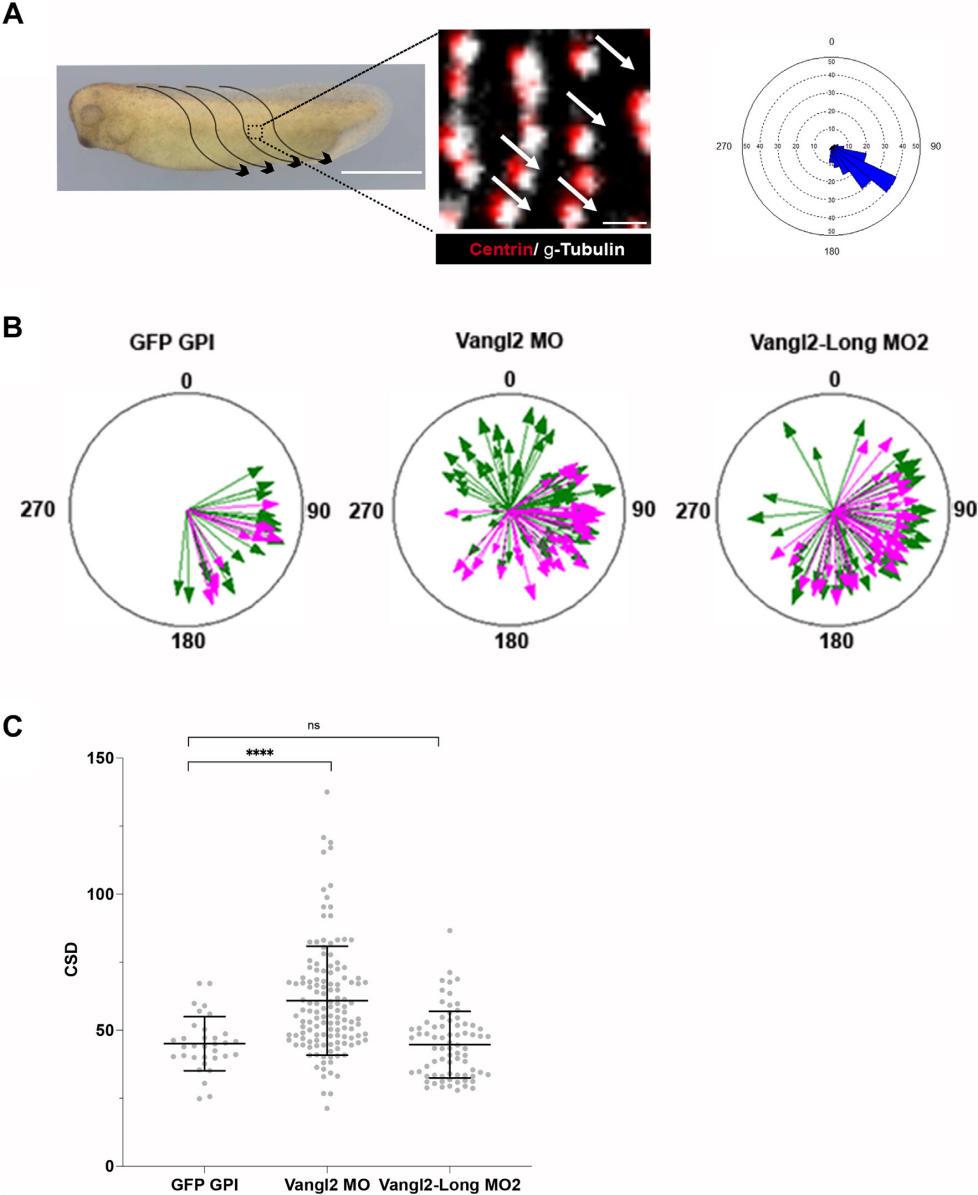
**Centriole polarization is impaired by Vangl2 and Vangl2-Long knockdown.***A*, rational of the experiment. The flow generated by the coordinated beating of myriads of cilia in epidermal MCCs is represented by *arrows* along the flanks of a stage 31 *Xenopus* embryo (the scale bars represent 1 mm). Centriole/cilium rotational polarity is evaluated through double IF staining of the basal body (BB, Centrin) and the basal foot (γ-Tubulin) (the scale bar represents 1 μm). For each MCC, a rose histogram is generated by the Oriana software to plot the angles of orientation of unambiguously doubly stained BBs. The coordinates of the rose plot are aligned on those of the embryo. *B*, *rose plots* displaying mean BB orientation in control, Vangl2, and Vangl2-Long morphant MCCs. Each *arrow* represents one MCC. The length of the *arrow* is proportional to the degree of alignment of BBs with the mean vector. The two colors represent MCCs from two independent experiments. Note that Vangl2 and Vangl2-Long knockdown cause the dispersion of angles of orientation of individual MCCs. *C*, *graph* displaying the circular SD (CSD) of the angles of orientation of BBs, within MCCs analyzed in *B*. A low CSD indicates that BBs are well aligned along the mean vector in a given MCC, whereas a high CSD indicates a poor degree of alignment. Note that CSD values were higher than control in Vangl2 but not in Vangl2-Long morphant MCCs, indicating poor BB alignment in the former case. Statistical analyses were carried out with GraphPad Prism software with a Student’s *t* test to evaluate significance. IF, immunofluorescence; MCC, multiciliated cell.

Next, we evaluated the importance of Vangl2 isoforms in centriole rotational polarity of *Xenopus* embryonic epidermal MCCs. To this end, we used double immunostaining to reveal the basal body (Centrin) and its associated asymmetric basal foot (γ-tubulin) (Fig. 6*A*) (37). For each basal body, a vector of orientation can thus automatically be drawn and all unambiguously identified vectors for a given MCC can be projected onto a rose plot, aligned on the polar coordinates of the embryo (Fig. 6*A*). In order to visualize multiple MCCs onto such plots, we used a mode of representation shared by other authors in the field, whereby each MCC is represented by an arrow, with a length proportional to the variation to the mean of basal body orientation vectors within that MCC (20, 22). Furthermore, a more precise measurement of the level of coordination of basal body orientation within individual cells was obtained by the calculation of the circular SD (CSD), with low CSD values indicating good alignment of orientation angles along the mean direction and high CSD values indicating poor alignment along the mean (Fig. 6*C*). In control embryos, most arrows point toward the ventroposterior quadrant of the rose plot, and are long, as few basal bodies deviate from the mean orientation within individual MCCs (Fig. 6*B*). In Vangl2 morphant embryos, many arrows were found to be smaller than controls (Fig. 6*B*), and CSD values were significantly higher in Vangl2 morphant *versus* control cells (Fig. 6*C*), reflecting a frequent deviation of basal body orientation from the mean direction within individual MCCs. In contrast, Vangl2-Long morphant MCCs displayed CSD values comparable to that of control cells (Fig. 6*C*), suggesting that intracellular coordination of basal body orientation was not significantly altered in this condition. However, many Vangl2-Long morphant MCCs projected outside the ventroposterior quadrant, consistent with a decrease of tissue-level coordination of orientation, typical of deficient PCP (20, 22). The same trend, albeit more severe, was observed in Vangl2 morphant embryos (Fig. 6*B*).

We conclude that both Vangl2 isoforms contribute to proper PCP deployment in the epidermis.

## Discussion

Here, we report on a previously uncharacterized Vangl2 isoform, Vangl2-Long, which we identified in human, mouse, and *Xenopus* and which bears an evolutionarily conserved N-terminal extension of 48 aa in human and mouse and 53 aa in *Xenopus*. In agreement with a previous bioinformatics study (34), our data establish that translation of the human VANGL2-Long isoform requires a near-cognate in frame AUA alternative translation initiation site located 144 nucleotides upstream of the canonical AUG start codon of VANGL2. In the cell lines tested so far, like in *Xenopus* embryos, the 70 kD Vangl2-Long isoform is found coexpressed with the canonical 62 kD Vangl2 protein and at low levels, possibly ascribing to the longer isoform a rate-limiting function. Unraveling the precise molecular mechanism at work in the choice of alternative translation initiation codons will be important to better understand the regulation of Vangl2 isoform expression.

Our findings that isoform-specific MOs against Vangl2-Long impair embryo morphogenesis, ciliated cell polarity and PCP molecule asymmetric distribution, provide strong indication that this longer isoform contributes a critical function to the PCP signaling pathway that control these events.

The capacity to encode an N-terminal extension is found in all Vangl2 loci analyzed so far and is absent in *Vangl1* transcripts implying that this feature has been acquired concomitantly with the gene duplication event that gave rise to these vertebrate paralogs. Vertebrates have a nervous system totally different from invertebrates as most of them, except hemichordate, urochordata, and cephalochorda, do not have a neural tube. The neural tube becomes a physiologically sealed system from a very early developmental stage, only in vertebrates (38). The addition of an N-terminal extension to Vangl2 therefore coincides with the acquisition of a neural tube specific function in vertebrates. Interestingly, fusing a tandem fluorescent tag to the endogenous murine *Vangl2* start codon has recently been reported to cause characteristic PCP phenotypes of varying penetrance (39). Depending on the zygosity status and genetic background, mild to severe morphogenetic defects have been observed in tdTomato-Vangl2 knockin mouse embryos, including curly tails, spina bifida, anencephaly or craniorachischisis. As mentioned by the authors, the genetically engineered *tdTomato-Vangl2* allele is predicted to disrupt the expression of Vangl2-Long, a molecular event that, based on the *Xenopus* data reported here, is likely to account for the neural tube closure defects observed in this mouse model. These observations reinforce our interpretation that Vangl2-Long, together with the short isoform, critically contributes to proper neural tube closure.

Along the same line, mutations in the human *VANGL1* and *VANGL2* genes have been linked to genetic diseases characterized by neural tube closure defects (12, 13). Given the PCP phenotypes elicited by loss of function of xVangl2-Long in *Xenopus* embryos, we wondered whether mutations associated with NTD or other developmental disorders related to PCP defects could map to the N-terminal extension of Vangl2-Long. However, genetic analysis of an NTD patient cohort failed to identify variants within the VANGL2-Long extension (E. Ross and V. Agular-Pulido, personal communications). One possibility is that mutations targeting this region may be hardly tolerated due to the functional importance of Vangl2-Long documented in this manuscript. Another possible explanation is that such mutations might be too infrequent to be detected in small cohorts. Testing these hypotheses will clearly require further investigations using larger cohorts of NTD patients.

Consistent with previous reports, we found that both overexpression and knockdown of Vangl2 in *Xenopus* embryos cause body axis shortening or severe bending and defective neural tube closure (18, 20, 21, 22). In *Drosophila*, either reduction or overexpression of Stbm (VANGL2 ortholog) causes characteristic PCP defects in the orientation of wing cell hairs (18). In zebrafish, overexpression and knockdown of Vangl2 disrupt CE movements associated with a body axis elongation defect, as well as abnormal neural tube closure (15). One possible explanation of these results is that the relative stoichiometry of Vangl1, Vangl2, and Vangl2-long in heterodimer and heterotrimer complexes (Figs. 2 and S3) must be fine-tuned to establish and maintain proper PCP in tissues. Accordingly, Strutt *et al.*, showed in *Drosophila* pupal wing epithelium that maintaining levels of Stbm molecules within some core PCP complexes is essential for strong asymmetry (40).

The capacity of VANGL paralogs to heterodimerize has been previously documented (11). The biochemical data presented here extend this property to the Vangl2-Long isoform in the human, mouse, and *Xenopus* contexts. The finding that the three VANGL proteins copurify as a tripartite multimeric complex echoes the recently described supramolecular organization of fly core PCP complexes in which up to six Vang/Stbm molecules assemble in signalosome-like structures (40). Here, we evaluated the molecular ratio between the Vangl2 and Vangl2-Long isoforms in IMCD3 cells through the quantification of the coimmunoprecipating 62 kD and 70 kD antigens that are both recognized by mAb 36E3. With this approach, we could estimate the Vangl2:Vangl2-Long ratio to range from 3:1 to 4:1 (Fig. S3). If one assumes the presence of one Vangl1 molecule per Vangl multimeric complex, it follows that the minimal Vangl1:Vangl2:Vangl2-Long subunit composition should vary from 1:3:1 to 1:4:1.

Intriguingly, we noticed in our immunoblots of *Xenopus* extracts, the presence of an additional band with an intermediate mobility, between xVangl2 and xVangl2-Long (see Figs. 3*A* and S5*A*). This intermediate band may stem from a posttranslational modification since its expression levels follow those of xVangl2 (Fig. 3*A*). Indeed, Vangl2 has been described to be phosphorylated in mouse cells (41). To address this possibility, protein extracts were treated with Lambda phosphatase, which caused the three bands to run faster (Fig. S7). This result suggests that the middle band represents a third Vangl2 isoform, which would be translated from another alternative site located between the *AUG* and the noncanonical *AUA* initiations codons. The observation that MOs against the long isoform decreased the intensity of this band (Fig. S5, *B* and *C*) also supports this view. Incidentally, our phosphatase assay reveals that all Vangl2 variants are phosphorylated in *Xenopus* embryos.

Altogether, these considerations reveal that while the molecular organization of Vang/Stbm and Vangl proteins appears to be conserved through evolution, the subunit composition has greatly evolved and increased in complexity with the appearance of multiple Vangl paralogs and isoforms.

Taking advantage of the cross-reactivity of mAb 36E3, we could analyze the distribution of endogenous Vangl2 proteins in both neural and epidermal cells of the *Xenopus* embryo. We could confirm the anterior enrichment of Vangl2 proteins in the plasma membrane of neural plate cells, reported in a previous study, which used a pAb also expected to detect both the canonical and long isoforms (35). Moreover, we report the first evidence of a polarized distribution of endogenous Vangl2 proteins in the posterior domain of the plasma membrane of mature epidermal cells. Previous studies reported that Vangl2 anterior localization in neural plate cells, as well as Vangl1 posterior localization in epidermal cells were both dependent on the proper activity of Pk2 (22, 35). Conversely, we show here that the posterior enrichment of Pk2 in epidermal cells is dependent on the presence of both Vangl2 and Vangl2-Long isoforms. Likewise, the anterior enrichment of Dvl1 in epidermal cells is also altered in Vangl2 and Vangl2-Long morphant embryos. This is consistent with the predominant view of mutually reinforcing interactions between Vangl and Pk proteins and mutually exclusive interactions with Fz and Dvl proteins, which help to partition cell membrane domains in embryonic tissues subjected to PCP.

Interestingly, we also noticed an enrichment of Vangl2 proteins in MCCs at the time of basal body docking and onset of ciliogenesis. Consistently, Vangl2 or Vangl2-Long knockdown impaired ciliogenesis, a phenotype also reported in zebrafish ependymal cells mutant for Vangl2 (42). Given the importance of ciliogenesis in development and health, the link to Vangl2 certainly deserves further investigation.

It has been shown for a variety of proteins that the use of non-AUG alternative initiation sites generates isoforms with distinct biochemical or cellular properties, providing putative mechanisms for further functional diversity (43). Whether the same applies to the Vangl2-Long isoform remains an open question. A recent study proposed distinctive and overlapping biological functions of RhoEα, a new Rho GTPase isoform generated from alternative translation, compared to the canonical RhoE (33). So far, our expression data as well as our functional evidence suggest that Vangl2 and Vangl2-Long have the same biochemical and cellular function. They are systematically coexpressed, predominantly polarized at the cell membrane, required for embryo morphogenesis and MCC polarity, and interchangeable for MCC ciliogenesis. The fact that the rather specific depletion of the Vangl2-Long isoform is sufficient to generate abnormal phenotypes suggests that it critically contributes, together with the short isoform, to generate a limited pool of Vangl2 molecules, necessary for PCP deployment. This idea is also supported by the partial depletion of both isoforms, caused by the morpholino directed against the canonical translation initiation site. It remains formally possible, however, that the N-terminal extension present in Vangl2-Long provides interactions with cytosolic components, which would be critical for PCP deployment by Vangl multimeric complexes. Several approaches, including yeast two-hybrid, peptide pull-down, or differential interaction screens, have been used in the aim of identifying such extension-specific protein interactors. However, all our attempts have remained unsuccessful so far, and we could not find any differences in terms of posttranslational modifications between Vangl2 and Vangl2-Long (data not shown). It will be therefore worthwhile addressing these mechanistic issues with alternative methods and by investigating the possible involvement of lipid-mediated interactions.

In summary, our findings provide a novel framework for the exploration of Vangl2 functions in PCP regulation through its multiple vertebrate isoforms.

## Experimental procedures

### Cell culture, transfections, and stable cell lines

Most cell lines used in this study were from and grown as recommended by American Type Culture Collection in the presence of 100 U/ml of penicillin, 100 μg/mlof streptomycin, and 10% heat-inactivated fetal bovine serum. KO-*VANGL2* HEK293T cells were kindly provided by Vita Bryja (Institute of Experimental Biology, Faculty of Science, Masaryk University). Murine epithelial IMCD3 cell line was cultured in Dulbecco's modified Eagle's medium/F12 growth medium, HEK293T cells were propagated in Dulbecco's modified Eagle's medium and SKBR7 cells were grown in Roswell Park Memorial Institute medium. Cell culture was performed at 37 °C in 5% CO2 incubator. A6 cells were grown at 27 °C in 55% Leibovitz’s L15 medium, 20 u/ml penicillin, 20 μg/ml streptomycin (Life technologies). For RNAi experiments, cells were transfected with 20 nM RNAi using Lipofectamine RNAiMAX reagent (Life technologies); cells were analyzed 48 or 72 h after RNAi treatment. We used PEI for transient transfection of DNA constructs in HEK293T cells. For stable cell lines, IMCD3 cells were transfected with DNA constructs using Lipofectamine Plus reagent (Life technologies) according to manufacturer’s recommendations. Stable transfectants were selected in medium containing 1 mg/ml geneticin (Life technologies) for 2 weeks. All cell lines were tested for *mycoplasma* and were free from *mycoplasma* contamination for all experiments.

### Generation of KO cells lines using CRISPR/Cas9

CRISPR-Cas9–mediated editing of *Vangl2* locus was achieved by generating a pSpCas9(BB)-2A-GFP (PX458, Addgene #48138) plasmid targeting the second exon of murine *Vangl2* (gRNA: 5′-TCGGCTATTCCTACAAGTC-3′). GFP-positive IMCD-3 cells were sorted by fluorescence activated cell sorting using an ARIA III (Becton Dickinson) cell sorter and individually seeded in 96-well plates. Clones were then expanded and screened by WB using mAb 36E3.

### Antibodies

Rat anti-VANGL2 antibodies (mAb 2G4 and mAb 36E3) were generated by immunizing rats with the cytoplasmic N-terminal region of VANGL2 as described elsewhere (11) N-VGL2 pAb was obtained by immunizing two rabbits with an 18-mer peptide sequence (PKRPQPAALERYKARRSD) spanning aa 30 to 47 of the N-terminal extension of VANGL2-Long. Immunoglobulins that specifically react against VANGL2-Long were obtained by affinity purification of N-VGL2 pAb on beads covalently coupled to the immunogenic peptide describesd above. Mouse anti-α-tubulin (T9026) was from Sigma. Mouse (JL-8) anti-GFP antibody was from TakaraBio.

Mouse anti-VANGL1 mAbs (mAb 3D1 and mAb 19D5) were produced in collaboration with Mimabs (Luminy-Marseille) by immunizing mice with a recombinant GST-tagged VANGL1 fragment spanning aa 1 to 102. Hybridoma supernatants were counter screened by ELISA against GST and GST-VANGL2. Clones that reacted specifically against GST-VANGL1 were selected for further biochemical and cytological characterization.

### Expression vectors and RNAi sequences

All expression plasmids used in this work are listed in Table S3. Detailed informations about their production can be obtained upon request. All RNAi targeting human sequences (siGENOME) and the nontargeting control RNAi (siCNT) were purchased from Dharmacon Inc. SiVANGL1#1 (5′GAACAUGAACGGCGAGUAA3′), SiVANGL2#2 (5′GGAAAUGAUUCUACUCGGA3′).

### WB analysis and IP assay

For WB analyses, cells were washed twice with ice-cold PBS and scraped immediately into ice-cold lysis buffer: 50 mM Hepes-NaOH (pH = 8), 150 mM NaCl, 10% glycerol, 2 mM EDTA and 0,5% NP-40 supplemented with a cocktail of protease inhibitors (Sigma-Aldrich). Cells were lysed during 15 min at 4 °C on a rotating wheel and centrifugated at 13,000 rpm during 30 min. Protein concentration was determined using a Bradford assay. Cell lysates were mixed with 4X Laemmli sample buffer and boiled for 5 min. Proteins samples were loaded on NuPAGE 4 to 12% Bis-Tris Gel (Life technologies) and separated by electrophoresis. Proteins were electrotransferred onto nitrocellulose blotting membranes (GE healthcare) and stained with Ponceau Red (Sigma-Aldrich). The membranes were blocked with Tris-buffered saline, 0.1% Tween 20, 5% (w/v) dried milk, incubated overnight with primary antibodies in blocking solution. Following extensive washing with Tris-buffered saline/0.1% Tween 20, the blots were incubated for 1 h with the appropriate secondary horseradish peroxidase-conjugated antibody (Life technologies). Horseradish peroxidase-mediated chemiluminescence was detected using an enhanced chemiluminescence reagent kits (GE Healthcare), and signals were quantified by densitometry, using ImageJ (https://imagej.net/ij/). WBs were reprobed to detect loading controls.

IP of GFP fusion proteins was done 48 h posttransfection. Thirty microliters of agarose beads covalently coupled to a GFP nanobody were prewashed with lysis buffer twice and incubated with 1 to 2 mg of lysates overnight at 4 °C on a rotating wheel. After extensive washing in lysis buffer, the bound material was eluted by the addition of Laemmli sample buffer 2X and boiled for 5 min at 95 °c. For IP of endogenous VANGL1, VANGL2, and VANGL2-Long, cell lysates were incubated overnight with indicated antibodies at 4 °C on a rotating wheel. The immune complexes were then precipitated with either protein A agarose beads (GE Healthcare) or G for 4 h at 4 °C, washed five times in lysis buffer, and analyzed by WB with the indicated antibodies.

### *Xenopus* embryo injections, plasmids, RNAs, and morpholinos

All experiments were performed following the Directive 2010/63/EU of the European parliament and of the council of 22 September, 2010 on the protection of animals used for scientific purposes and approved by the “Direction départementale de la Protection des Populations, Pôle Alimentation, Santé Animale, Environnement, des Bouches du Rhône” (agreement number G 13055 21).

Eggs obtained from WT *X. laevis* females of 2 to 5 years of age (NASCO, https://www.enasco.com) were fertilized *in vitro* with sperm from NASCO males, dejellied, and cultured as described previously (44). After injection, embryos were incubated at 13 °C, 18 °C, or 23 °C in 0.1x Modified Barth's Solution until they reached the desired developmental stage.

pCS-GFP-Vangl2, Dvl1-eGFP, and eGFP-Pk2 plasmids were kindly provided by J.B. Wallingford. The pGFP-Vangl2-Long and pRFP-Vangl2-Long described in Table S3 have been generated by PCR with a series of overlapping extended primers reconstituting the 159 nucleotide sequence upstream of the canonical AUG codon of xVangl2 (further informations available upon request). Synthetic mRNAs were produced using Ambion mMESSAGE mMACHINE Kit with Sp6 polymerase and purified with Macherey-Nagel NucleoSpin RNA Clean-up kit. 100 pg of mRFP-capped mRNA was used as injection control and tracer. 1000 pg each of GFP-xVangl2 and GFP-xVangl2-Longcapped mRNAs were injected for overexpression in the two dorsal blastomeres at 4-cell stage. 40 pg each of GFP-xVangl2 and GFP-xVangl2-Long–capped mRNAs were injected to assess cellular localization in live embryos.

Morpholino antisense oligonucleotides were obtained from Genetools:

Standard Control MO by Genetools: 5′-CCTCTTACCTCAGTTACAATTTATA-3′

Vangl2 MO: 5′-ACTGGGAATCGTTGTCCATGTTTC-3′ (20) Two independent morpholinos antisense oligonucleotides were designed against *xVangl2-Long* mRNA:

Vangl2- Long MO(1): 5′-CAACTTTCCTTTAGCGACTCTAT-3′

Vangl2-Long MO(2): 5′-TAGCGACTCTATTTTGATTGGCTGT-3′

MO(1) was designed to bind 23 bases starting at the ATA noncanonical start codon, and MO(2) was designed to bind 25 bases starting at position −13 from the ATA codon.

Embryos at 2- or 4-cell stage were injected four times in the marginal zone with the following doses of MOs (Vangl2 MO = 30 ng; Vangl2-Long MO(1) = 50 ng; Vangl2-Long MO(2) = 40 ng) for morphological analysis and at 8- or 16-cell stage in the two ventral blastomeres with half of the above-mentioned doses for the other analyses.

100pg GFP-Pk2 and 50pg Dvl1-GFP were injected in one ventral blastomere at 16-cell stage with or without morpholinos and mRFP.

### Immunostaining and confocal microscopy of *Xenopus* embryos

Embryos were fixed at stage 16 or 31 in paraformaldéhyde 4% in PBS-Triton 0.1% during 30 min at room temperature and blocked in bovine serum albumine 3% in PBS for 1 h at room temperature. Incubation with the primary antibodies were done O/N at 4 °C at the following concentrations: chicken anti-GFP (Aves GFP-1020, 1/500), rabbit anti-RFP (600401379 Rockland, 1:500), rat anti-Vgl2 (36E3) homemade (1:5000), mouse anti-ZO1-TJP1 (Thermo Fisher Scientific 33-9100, 1:200), mouse anti-γ-tubulin (Abcam Ab 11316, 1:800), mouse anti-acetylated-tubulin (Sigma T7451, 1/1000), and mouse anti-Centrin (Millipore cat#04-1624, 1:500). Incubation in secondary antibodies were done 1 h at 4 °C with alpaca anti-mouse IgG1-647 (Life Technologies, 1:1000), anti-rat-488 (Life Technologies, 1:800), anti-rabbit-568 (Life Technologies, 1:800), goat anti-mouse IgG2b-647 (Life Technologies, 1:800), goat anti-mouse IgG2a-568 (Life Technologies, 1:800), and anti-chicken-488 (Jackson ImmunoResearch, 1:800). Embryos were washed in PBS, mounted in Mowiol 4-88 (SIGMA # 81381), and imaged with a ZEISS LSM 780 right standing AxioImager Z2 using a 40x objective and analyzed using ImageJ/FIJI (https://imagej.net/ij/) and Photoshop (Adobe; https://www.adobe.com/) softwares.

To evaluate Pk2-GFP and GFP-Dvl1 asymmetric localization at the plasma membrane, acquisitions were done on live embryos, mounted in Fluorogel (EMS CAT.17985-10) between slide and coverslip (45) with a ZEISS LSM 780 right standing AxioImager Z2 40x objective, and Z stacks in maximum intensity projection were analyzed using ImageJ/FIJI. Quantification of fluorescence intensity was based on the method reported in (22), and the results were analyzed with the GraphPad Prism software (https://www.graphpad.com/) with a Student’s *t* test for the significance.

To quantify cilia orientation, stage 31 fixed embryos were immunostained against Centrin to detect basal bodies (BB) and against γ-tubulin to detect basal feet. Basal body orientation was analyzed using a home-made ImageJ script (designed by R. Flores-Flores) (37). The output of the script is a list of angles that were plotted using the Oriana software (Version 4.02, Kovach Computing Services; https://www.kovcomp.co.uk/oriana/) to obtain a graphical representation of their distribution. CSD were plotted and analyzed with the GraphPad Prism software with a Student’s *t* test for the significance. In rose plots, each arrow represents the mean vector of a single MCC, and its length represents the significance of the mean vector; each color represents one independent experiment.

### MS analysis

Immunoprecipitated proteins were loaded on NuPAGE 4 to 12% Bis-Tris acrylamide gels (Life Technologies) to stack proteins in a single band that was stained with Imperial Blue (Pierce) and cut from the gel. Gels pieces were submitted to an in-gel trypsin digestion. Briefly, gel pieces were washed and destained using 100 mM NH4HCO3/acetonitrile (50/50). Destained gel pieces were shrunk with acetonitrile and were reswollen in the presence of 100 mM ammonium bicarbonate in 50% acetonitrile and dried at room temperature. Protein bands were then rehydrated and cysteines were reduced using 10 mM DTT in 100 mM ammonium bicarbonate pH 8 for 45 min at 56 °C before alkylation in the presence of 55 mM iodoacetamide in 100 mM ammonium bicarbonate pH 8 for 30 min at room temperature in the dark. Proteins were then washed twice in 100 mM ammonium bicarbonate and finally shrunk by incubation for 5 min with 100 mM ammonium bicarbonate in 50% acetonitrile. The resulting alkylated gel pieces were dried at room temperature. The dried gel pieces were reswollen by incubation in 100 mM ammonium bicarbonate pH 8 supplemented with trypsin (12.5 ng/μl; Promega) for 1 h at 4 °C and then incubated overnight at 37 °C. Peptides were harvested by collecting the initial digestion solution and carrying out two extractions; first in 5% formic acid and then in 5% formic acid in 60% acetonitrile. Pooled extracts were dried down in a centrifugal vacuum system. Samples were reconstituted with 0.1% trifluoroacetic acid in 4% acetonitrile and analyzed by LC-MS/MS using an Orbitrap Fusion Lumos Tribrid Mass Spectrometer (Thermo Electron) both online with a nanoRSLC Ultimate 3000 chromatography system (Dionex). Peptides were separated on a Dionex Acclaim PepMap RSLC C18 column. For peptide ionization in the EASY-Spray nanosource in front of the Orbitrap Fusion Lumos Tribrid Mass Spectrometer, spray voltage was set at 2.2 kV and the capillary temperature at 275 °C. The Orbitrap Lumos was used in data-dependent mode to switch consistently between MS and mass spectrometry/mass spectrometry (MS/MS). Time between Masters Scans was set to 3 s. MS spectra were acquired with the Orbitrap in the range of *m/z* 400 to 1600 at a full width at half maximum resolution of 120,000 measured at 400 *m/z*. AGC target was set at 4.0e5 with a 50 ms maximum injection time. For internal mass calibration the 445.120025 ions was used as lock mass. The more abundant precursor ions were selected and collision-induced dissociation fragmentation was performed in the ion trap to have maximum sensitivity and yield a maximum amount of MS/MS data. Number of precursor ions was automatically defined along run in 3 s windows using the “inject ions for all available parallelizable time option” with a maximum injection time of 300 ms. The signal threshold for an MS/MS event was set to 5000 counts. Charge state screening was enabled to exclude precursors with 0 and 1 charge states. Dynamic exclusion was enabled with a repeat count of 1 and a duration of 60 s.

### Data processing protocol

Raw files generated from MS analysis were processed with Proteome Discoverer 1.4 .1.14 (Thermo Fisher Scientific) to search against the proteome reference of the *X. laevis* protein database (56,145 entries, extracted on February 2018). The original fasta file was implemented with the long Vangl2 isoform by adding to the Nterminus part of the vangl2A sequence (accession number: Q90X64) the following 53 aa IESLKVKVDFLKVPFGLKKPVLKEAVAVLASTQGSGGPKSANVDRHKSRYSEN-. Database search with SequestHT were done using the following settings: a maximum of two trypsin miss cleavage allowed, methionine oxidation and N terminal protein acetylation as variable modifications and cysteine carbamidomethylation as fixed modification. A peptide mass tolerance of 6 ppm and a fragment mass tolerance of 0.8 Da were allowed for search analysis. Only peptides with high Sequest scores were selected for protein identification. False discovery rate was set to 1% for protein identification.

## Data availability

All data generated in this study are included in this article and the Supporting information.

## Supporting information

This article contains supporting information.

## Conflict of interest

The authors declare that they have no conflicts of interest with the contents of this article.
