# Supplementary material for: A vertebrate Vangl2 translational variant required for planar cell polarity
Source: J Biol Chem. 2024 Feb 24;300(4):106792. doi: 10.1016/j.jbc.2024.106792 (PMC11065751; doi:10.1016/j.jbc.2024.106792)
Supplement: Supporting Figures [file mmc1.pdf]

A

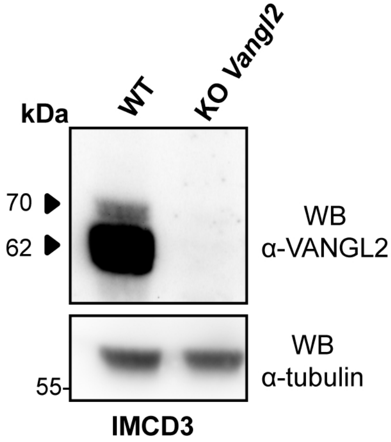

WT
KO Vangl2
kDa
70
62
WB
α-VANGL2
WB
α-tubulin
55
IMCD3

B

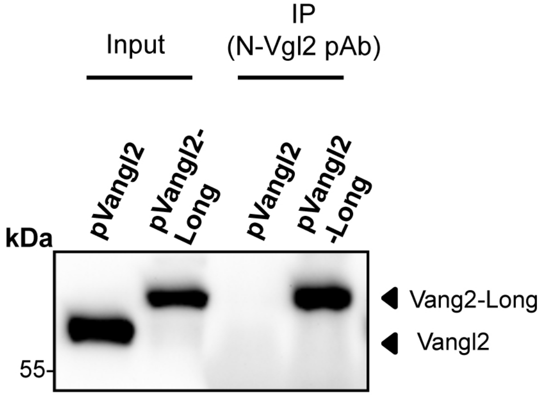

Input
IP
(N-Vgl2 pAb)
pVangl2
pVangl2-Long
pVangl2
pVangl2-Long
kDa
Vang2-Long
Vangl2
55

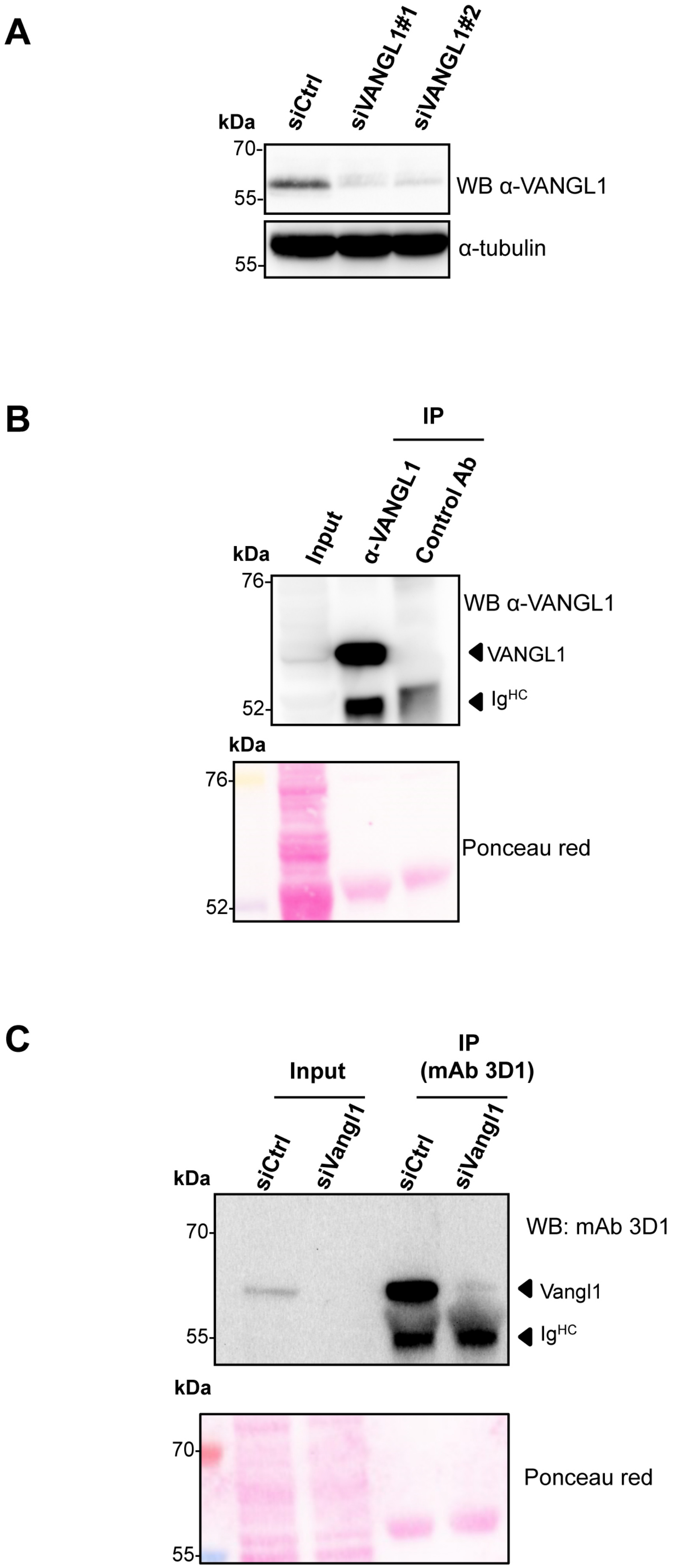
A
kDa
siCtrl
siVANGL1#1
siVANGL1#2
70
55
WB α-VANGL1
55
α-tubulin
B
IP
kDa
Input
α-VANGL1
Control Ab
76
52
WB α-VANGL1
VANGL1
Ig^HC
kDa
76
52
Ponceau red
C
Input
IP
(mAb 3D1)
kDa
siCtrl
siVangl1
siCtrl
siVangl1
70
55
WB: mAb 3D1
Vangl1
Ig^HC
kDa
70
55
Ponceau red

A

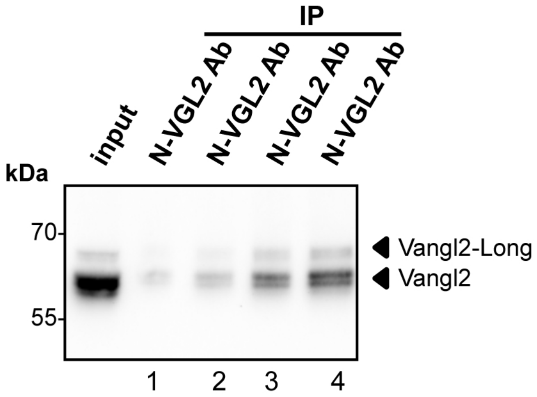

B

	Vangl2-Long	Vangl2	Ratio
**Input**	3587,79	30761,146	**8,57**
**1**	658,678	2315,619	**3,51**
**2**	1154,92	4775,154	**4,13**
**3**	3076,426	12468,782	**4,05**
**4**	5080,326	19531,953	**3,8**

C

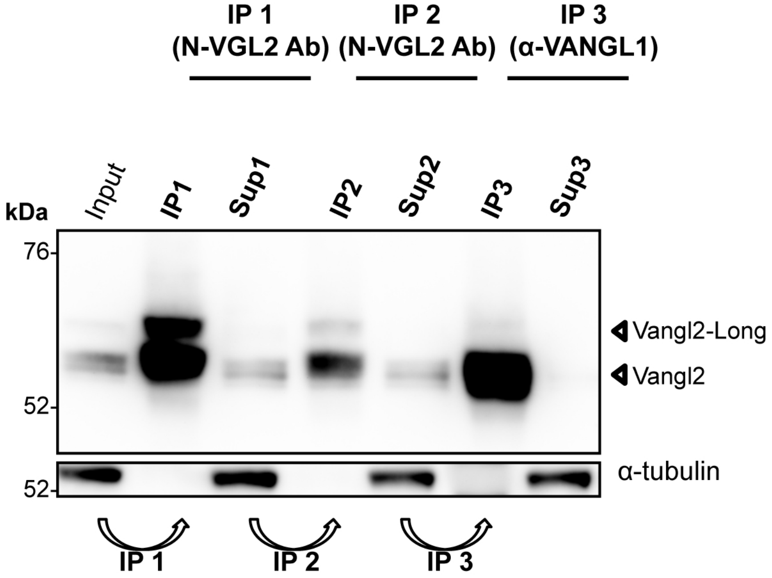

A

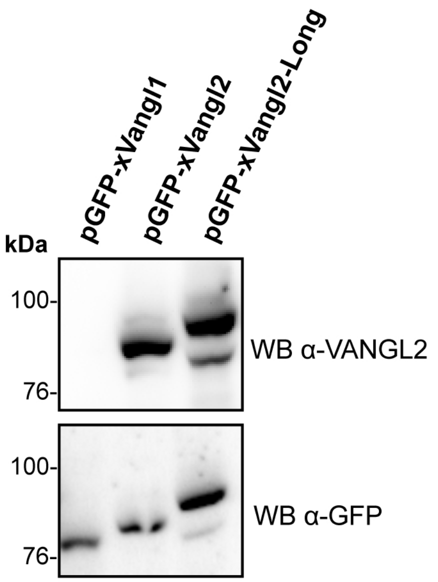

B

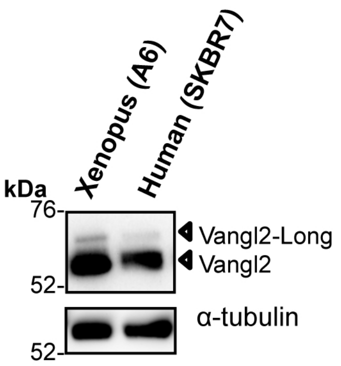

C

```
                                                    MDNDSQYSGYSYKS vangl2.S/L
IESLKIKVDFLKVPFGLKKPVLKEAVAVLASTQGSGGPKPANLDRHKSRYSENMDNDSQYSGYSYKS vangl2.S
IESLKVKVDFLKVPFGLKKPVLKEAVAVLASTQGSGGPKSANVDRHKSRYSENMDNDSQYSGYSYKS vangl2.L

GQSRSSRKHRDRRERHRSKSREGSRGDKSVTIQAPGEPLLDNESTRGEDRDDNWGETTTVVTGTSEH vangl2.S
GHSRSSRKHRDRRERHRSKSREGSRGDKSVTIQAPGEPLLDNESTRGEDRDDNWGETTTVVTGTSEH vangl2.L

SISHDDITRITKDMEDSAKLDCSRHLGVVIAGALALLSFLTPIAFMLLPQILWREDLEQCGTACEGL vangl2.S
SISHDDITRITKDMEDSAKLDCSRHLGVVIAGALALLSFLTPIAFMLLPQILWREDLEQCGTACEGL vangl2.L

FISVAFKLLILLLGSWALFFRRPKAFFPRVFVFRALLMV LVFLLVVSYWLFYGVRILESRDKNYQGI vangl2.S
FISVAFKLLILLLGSWALFFRRPKAFFPRVFVFRALLMV LVFLLVVSYWLFYGVRILESRDKNYQGI vangl2.L

VQYAVSLVDALL FVHYLAVVLLELRQLQPQFTIKVVRSTDGASRFYNIGHLSIQRVAVWILENYYHD vangl2.S
VQYAVSLVDALL FVHYLAVVLLELRQLQPQFTIKVVRSTDGASRFYNIGHLSIQRVAVWILENYYHD vangl2.L

FPVYNPALLNLPKSILSKKMSGFKVYSLGEENNTNNSTGQSRAVIAAAARRRDNSHNEYYYEEAEHE vangl2.S
FPVYNPALLNLPKSILSKKMSGFKVYSLGEENTTNNSTGQSRAVIAAAARRRDNSHNEYYYEEAEHE vangl2.L

RRVRKRKARLVVAVEEAFTHIKRLQDEDPKNPREIMDPREAAQAIFASMARAMQKYLRTTKQQPYHT vangl2.S
RRVRKRKARLVVAVEEAFTHIKRLQDEDQKNPREIMDPREAAQAIFASMARAMQKYLRTTKQQPYHT vangl2.L

MESILQHLEFCITHDMTPKAFLERYLGPGPTIQYHKDRWLAKQWTLVSEEPVTNGLKDG VVFELKRQ vangl2.S
MESILQHLEFCITHDMTPKAFLERYLGPGPTIQYHKDRWLAKQWTLVSEEPVTNGLKDG VVFVLKRQ vangl2.L

DFSLVVSTKKIPFFKLSEEFVDPKSHKFVMRLQSETSV vangl2.S
DFSLVVSTKKIPFFKLSEEFVDPKSHKFVMRLQSETSV vangl2.L
```

D

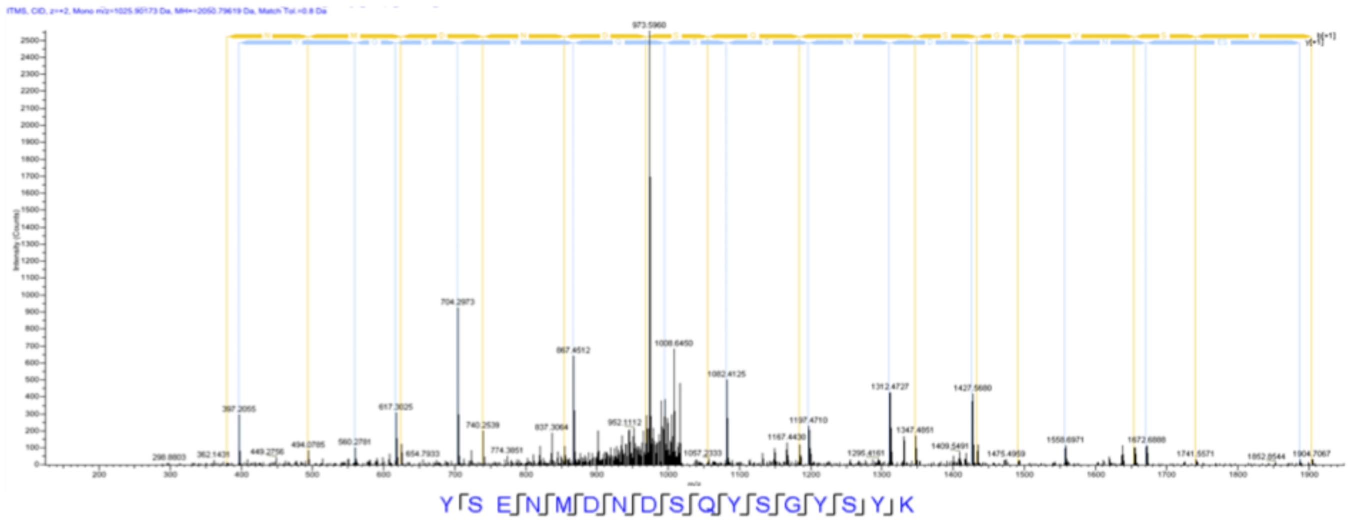

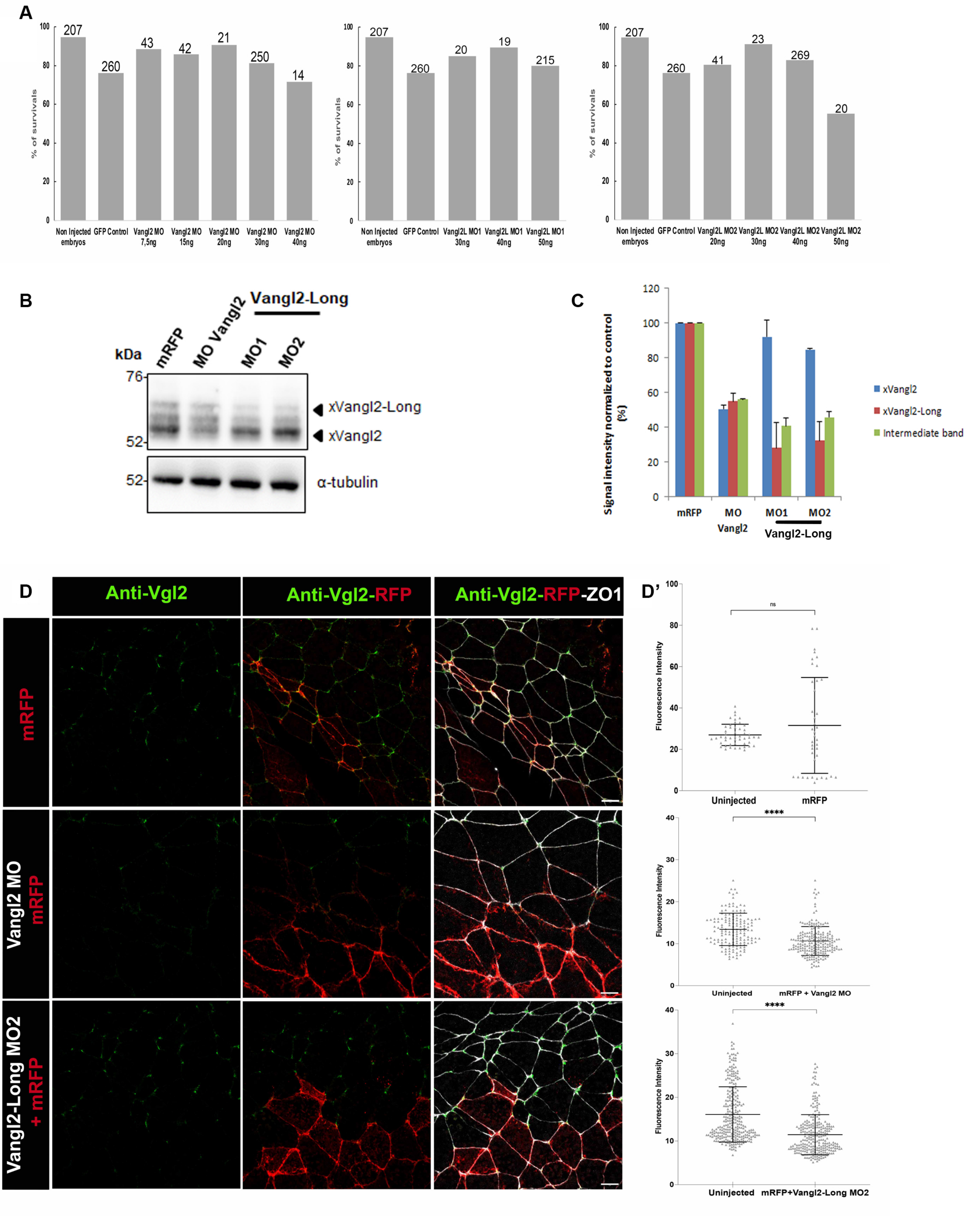
A
% of survivals
207
260
43
42
21
250
14
Non Injected embryos
GFP Control
Vangl2 MO 7,5ng
Vangl2 MO 15ng
Vangl2 MO 20ng
Vangl2 MO 30ng
Vangl2 MO 40ng
207
260
20
19
215
Vangl2L MO1 30ng
Vangl2L MO1 40ng
Vangl2L MO1 50ng
207
260
41
23
269
20
Vangl2L MO2 20ng
Vangl2L MO2 30ng
Vangl2L MO2 40ng
Vangl2L MO2 50ng
B
kDa
mRFP
MO Vangl2
Vangl2-Long
MO1
MO2
76
52
52
xVangl2-Long
xVangl2
α-tubulin
C
Signal intensity normalized to control (%)
xVangl2
xVangl2-Long
Intermediate band
mRFP
MO Vangl2
MO1
MO2
Vangl2-Long
D
Anti-Vgl2
Anti-Vgl2-RFP
Anti-Vgl2-RFP-ZO1
mRFP
Vangl2 MO mRFP
Vangl2-Long MO2 + mRFP
D'
Fluorescence Intensity
ns
Uninjected
mRFP
****
mRFP + Vangl2 MO
****
mRFP+Vangl2-Long MO2

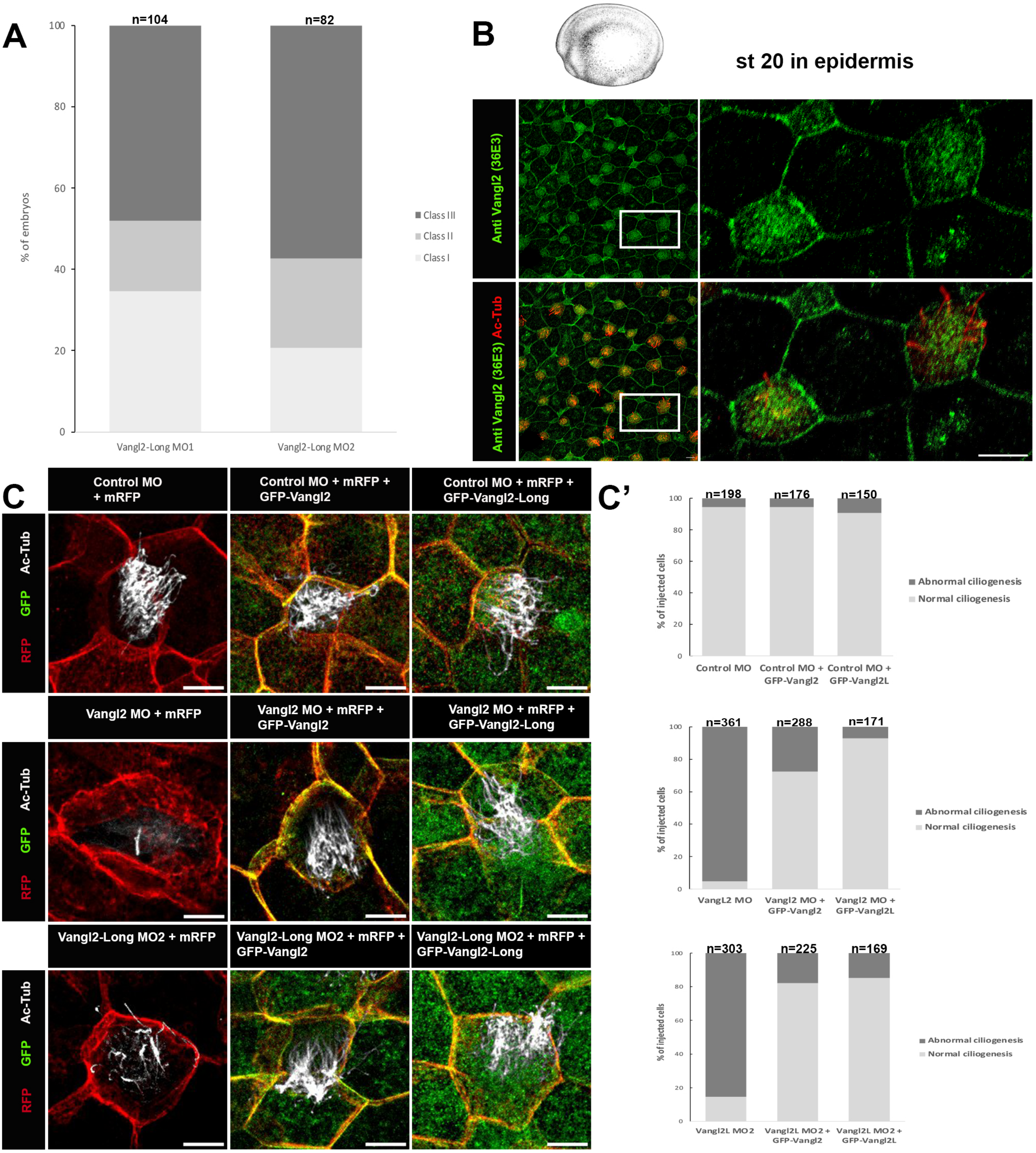
A
n=104
n=82
% of embryos
Class III
Class II
Class I
Vangl2-Long MO1
Vangl2-Long MO2
B
st 20 in epidermis
Anti Vangl2 (36E3)
Anti Vangl2 (36E3) Ac-Tub
C
Control MO + mRFP
Control MO + mRFP + GFP-Vangl2
Control MO + mRFP + GFP-Vangl2-Long
Vangl2 MO + mRFP
Vangl2 MO + mRFP + GFP-Vangl2
Vangl2 MO + mRFP + GFP-Vangl2-Long
Vangl2-Long MO2 + mRFP
Vangl2-Long MO2 + mRFP + GFP-Vangl2
Vangl2-Long MO2 + mRFP + GFP-Vangl2-Long
RFP GFP Ac-Tub
C'
n=198
n=176
n=150
% of injected cells
Abnormal ciliogenesis
Normal ciliogenesis
Control MO
Control MO + GFP-Vangl2
Control MO + GFP-Vangl2L
n=361
n=288
n=171
VangL2 MO
Vangl2 MO + GFP-Vangl2
Vangl2 MO + GFP-Vangl2L
n=303
n=225
n=169
Vangl2L MO2
Vangl2L MO2 + GFP-Vangl2
Vangl2L MO2 + GFP-Vangl2L

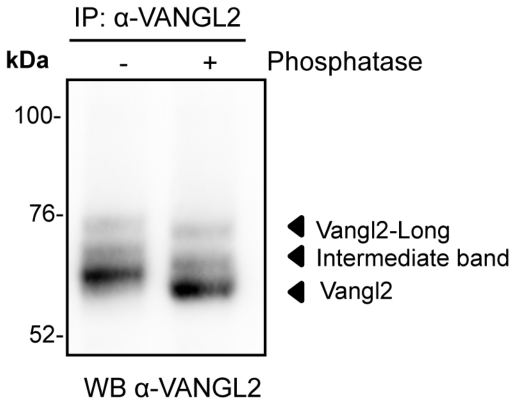
IP: α-VANGL2
kDa
-
+
Phosphatase
100-
76-
52-
Vangl2-Long
Intermediate band
Vangl2
WB α-VANGL2
