# Supplementary material for: A vertebrate Vangl2 translational variant required for planar cell polarity
Source: J Biol Chem. 2024 Feb 24;300(4):106792. doi: 10.1016/j.jbc.2024.106792 (PMC11065751; doi:10.1016/j.jbc.2024.106792)
Supplement: Supporting Tables [file mmc2.pdf]

Sequence	Vangl2 isoform (1)	Position in Vangl2A/2B sequence	MH+ (Da) (2)	PSM number (3)	Xcorr Interval	PEP (4)
YSENMDNDSQYSGYSYK	*Extension*	*-4-13*	*2050.79619*	*5*	*3.96-5.05*	2.15 e^{-8}
Ac-MDNDSQYSGYSYK	Vangl2A/B	1-13	1599.62883	2	3.21-3.48	0.00552
SVTIQAPGEPLLDNESTR	Vangl2A/B	43-60	1926.97417	6	3.58-4.11	0.0229
GEDRDDNWGETTTVVTGTSEHSISHD DITR	Vangl2A/B	61-90	3330.47207	2	3.33-3.42	0.0125
FYNIGHLSIQR	Vangl2A/B	259-270	1347.71665	3	1.35-2.27	0.0791
VYSLGEEN**N**TNNSTGQSR	Vangl2B	306-324	1969.88432	6	3.73-4.40	1.23 e^{-6}
VYSLGEEN**T**TNNSTGQSR	Vangl2A	306-324	1969.88432	3	3.38-3.67	0.00204
RDNSHNEYYYEEAEHER	Vangl2A/B	334-351	2240.91982	6	4.31-4.44	6.06 e^{-7}
DNSHNEYYYEEAEHER	Vangl2A/B	335-351	2084.82207	7	3.66-4.34	0.00236
DNSHNEYYYEEAEHERR	Vangl2A/B	335-352	2240.92153	3	2.77-3.33	0,00542
LVVAVEEAFTHIK	Vangl2A/B	358-371	1455.81999	3	2.56-3.31	0,000144
LQDED**P**KNPR	Vangl2B	373-382	1211.60149	2	2.34-2.49	0.169
RLQDED**Q**KNPR	Vangl2A	372-382	1211.60149	1	3.05	0.000112
EAAQAIFASMAR	Vangl2A/B	388-400	1281.62358	10	3.80-4.22	0.0235
YLGPGPTIQYHK	Vangl2A/B	441-452	1373.72110	1	1.44	0.26
QWTLVSEEPVTNGLK	Vangl2A/B	458-473	1700.88530	3	2.64-3.39	3.47 e^{-5}
DGVVF**E**LK	Vangl2B	474-481	906.49187	3	1.64 - 2.27	0.00511
DGVVF**V**LK	Vangl2A	474-481	906.49187	6	2.27-2.44	0.00899
RQDFSLVVSTK	Vangl2A/B	482-492	1279.69756	2	2.34-2.56	0.002
LSEEFVDPK	Vangl2A/B	498-507	1063.53081	3	2.36-2.84	0.00498

Table S1

Run1

Accession	Description	TOP3 AREA (1)	Sequest Score	Sequence Coverage	# Peptides (2)	# PSM (3)
Q90Z05/Q90X64	Vang-like protein 2-A/2-B (4)	9,379E7	62,20	35,70	15	22
A0A1L8H5Y3	Vang-like protein 1	2,567E6	10,40	10,74	4	4

Run2

Accession	Description	TOP3 AREA	Sequest Score	Sequence Coverage	# Peptides	# PSM
Q90Z05/Q90X64	Vang-like protein 2-A/2-B	1,331E8	70,65	38,00	16	23
A0A1L8H5Y3	Vang-like protein 1	2,833E6	10,71	10,74	4	4

Run3

Accession	Description	TOP3 AREA	Sequest Score	Sequence Coverage	# Peptides	# PSM
Q90Z05/Q90X64	Vang-like protein 2-A/2-B	8,495E7	51,62	23,42	11	17
A0A1L8H5Y3	Vang-like protein 1	2,961E6	10,91	10,74	4	4

Table S2

Plasmid name	insert	origin	cloning method
pVangl2	human Vangl2 (aa 1-521) ORF	this study	Gateway
pVangl2-SL	human Vangl2 (aa 1-521) ORF + 342bp 5'UTR	this study	Gateway
pVangl2-Long	human Vangl2 (aa 1-521) ORF + 144bp 5'UTR (ATA^{-144} -> ATG and ATG^{1} -> GCA)	this study	Gateway
pGFP-Vangl2	human Vangl2 (aa 1-521) ORF cloned downstream of GFP	Belotti et al., 2012	Gateway
pGFP-Vangl2-Long	human Vangl2 (aa 1-521) ORF + 144bp 5'UTR cloned downstream of GFP	this study	Gateway
pGFP-Vangl1	human Vangl1 (aa 1-524) ORF cloned downstream of GFP	Belotti et al., 2012	Gateway
pVangl2-SL-ATA-to-GCA	human Vangl2 (aa 1-521) ORF + 342bp 5'UTR ATA^{-144} -> GCA	this study	Gateway
pVangl2-SL-ATG-to-GCA	human Vangl2 (aa 1-521) ORF + 342bp 5'UTR ATG^{1} -> GCA	this study	Gateway
pGFP-Vangl1 (Xenopus)	Xenopus laevis Vangl1 ORF	J. Wallingford	
pGFP-Vangl2 (Xenopus)	Xenopus laevis Vang2 ORF	J. Wallingford	
pRFP-Vangl2 Long (Xenopus)	Xenopus laevis Vang2 ORF + 159bp 5'UTR	this study	Not1-Xho1 fragment
pCS-GFP-Vangl2 Long (Xenopus)	Xenopus laevis Vangl2 ORF + 159bp 5'UTR	this study	Not1-Xho1 fragment

Table S3
