# Supplementary material for: A vertebrate Vangl2 translational variant required for planar cell polarity
Source: J Biol Chem. 2024 Feb 24;300(4):106792. doi: 10.1016/j.jbc.2024.106792 (PMC11065751; doi:10.1016/j.jbc.2024.106792)
Supplement: Supporting Information [file mmc3.docx]

**Figure S1. Validation of anti-VANGL2 (mAb 36E3) and anti-Vangl2-Long (N-VGL2 pAb) antibodies. (A)** IMCD3 cells with either a wild type (WT Vangl2) or Crispr-Cas9 generated knockout (KO *Vangl2*) *Vangl2* alleles were assessed for Vangl2 expression by Western blot analysis with mAb 36E3. Note that Crispr-Cas9 mediated knockout of *Vangl2* abrogates the expression of both the 62 kD and 70 kD proteins detected by mAb 36E3. **(B)** KO *Vangl2* IMCD3 cells stably transfected with pVANGL2 or pVANGL2-long were subjected to immunoprecipitation with N-VGL2 pAb before Western blot analysis with mAb 36E3. Note the ability of N-VGL2 pAb to immunoprecipitate VANGL2-Long but not VANGL2.

**Figure S2. Validation of two VANGL1 specific monoclonal antibodies, mAb 19D5 and 3D1 which detect human VANGL1 and mouse Vangl1 respectively (A)** MCF7 cells were treated with two independent siRNAs directed against human VANGL1 (siVANGL1#1 and siVANGL1#2) or a non-targeting siRNA (siCtrl). Total cell lysates were then analyzed by Western blotting with a novel VANGL1 specific monoclonal antibody, mAb 19D5, or with an anti-α tubulin used as a loading control. **(B)** Immunoprecipitation of MCF-7 cell extracts with mAb 19D5 or a mouse isotypic control antibody (Control Ab). Protein IPs were analyzed by WB with the same mAb19D5 antibody. The lower panel shows an image of the transfer membrane stained with Ponceau red. **(C)** Murine epithelial (IMCD3) cells treated with VANGL1 siRNA (siVANGL1) or a non-targeting siRNA (siCtrl) were processed for immunoprecipitation using a novel VANGL1 monoclonal antibody, mAb 3D1. Immunoprecipitated proteins were probed by WB with the same antibody. Arrows indicate the presence of the endogenous murine VANGL1 protein and the immunoglobulin heavy chain recognized by the secondary anti-mouse antibody. The lower panel shows an image of the transfer membrane stained with Ponceau red in order to confirm equal loading of each control and experimental samples.

**Figure S3.** **The Vangl2:Vangl2-Long ratio range from 3:1 to 4:1 in IMCD3 cells.**
**(A)** IMCD3 cell extract was immunoprecipitated with N-VGL2 pAb. Increasing amounts of the same immunoprecipitate (from lane 1 to lane 4 : 0,5/3 - 1/3 - 1/2 - 2/3 of the immunoprecipitate) were probed for the presence of Vangl2 and Vangl2-Long by Western blotting with mAb 36E3. **(B)** Quantification of the Vangl2:Vangl2-Long ratio as indicated. **(C)** Evidence for the existence of bi- and tri-partite complexes between Vangl1, Vangl2 and Vangl2-Long in IMCD3 cells. An IMCD3 cell extract was successively immunoprecipitated twice with Vangl2-Long-specific immunoglobulins (N-VGL2 Ab, IP1 and IP2) before subjecting the Vangl2-Long-depleted lysate (N-VGL2 Ab/Sup2, lane 5) to a third immunoprecipitation step using the VANGL1-specific antibody mAb3D1 (IP3/α-VANGL1, lane 6). All fractions, including the original lysates and the three successive IPs and supernatants were probed for the presence of Vangl2 and Vangl2-Long by Western blotting with mAb36E3. The same immunoblot was subsequently probed for α-tubulin that served as a loading control. Note the enrichment of Vangl2 and Vangl2-Long in the IP1 sample (IP1, lane 2) and the depletion of Vangl2-Long after the first (Sup1, lane 3) and second round of immunoprecipitation with N-VGL2 Ab (Sup2, lane 5). Also note that the pool of Vangl2 not immunoprecipitated by N-VGL2 Ab was efficiently co-precipitated with Vangl1 in the α-Vangl1 IP3 sample (IP3, lane 6)

**Figure S4. Biochemical characterization of xVangl2-Long in *Xenopus* embryos.** **(A)** IMCD3 cells transfected with GFP-tagged *Xenopus* Vangl1 (GFP-xVangl1), Vangl2 (GFP-xVangl2) and Vangl2-Long (GFP-xVangl2-Long) constructs were probed by Western blotting with mAb 36E3 (upper panel) or anti-GFP antibodies (lower panel). Note the specific immunoreactivity of mAb 36E3 against the GFP fusions of xVangl2 and xVangl2-Long proteins but not xVangl1. **(B)** Western blot analysis of *Xenopus* A6 and human SKBR7 cells using mAb 36E3. lmmunoblotting with an anti-tubulin antibody is shown in the lower panel and can be used as a protein loading control. Note the detection by mAb 36E3 of the 62 kD Vangl2 and the 70 kD Vangl2-Long polypetides in both samples. **(C)** Peptide coverage of xVangl2 as determined by mass spectrometry analysis. Sequences (in cyan) corresponding to both Vangl2.S (formerly Vangl2B, upper) and Vangl2.L (formerly Vangl2A, lower) *Xenopus* homeologs were detected by LC-MSMS. Slight differences in amino acids are indicated in red. Both the canonical N-terminal sequence MDNDSQYSGYSYK and an N-terminally extended sequence YSENMDNDSQYSGYSYK were identified, and are indicated in green. **(D)** ms/ms spectrum of the YSENMDNDSQYSGYSYK peptide MH+: 2050.7961 Da.

**Figure S5. Characterization of Vangl2 and Vangl2-Long morpholinos.** **(A)** Quantification of the survival rate of injected embryos in response to increasing doses of Vangl2, Vangl2-Long MO1 and Vangl2-Long MO2 morpholinos. (**B**) *Xenopus* embryos were injected with synthetic mRNA encoding mRFP as control, or with a MO directed against Vangl2 (Vangl2 MO), or two independent MOs targeting Vangl2-Long (Vangl2-Long MO1 and MO2) in dorsal blastomeres at 2-cell stage and grown until stage 13. Western blot analysis was carried out with mAb 36E3 (upper panel) and anti-tubulin as loading control (lower panel). (**C**) Quantification of xVangl2 (blue), xVangl2-Long (red) and intermediate band (green) signals detected in (A), normalized to tubulin loading control in two independent experiments. Note the efficient knockdown of xVangl2-Long achieved with the two independent MOs directed against this isoform, while xVangl2 expression was largely unaffected. (**D**) *Xenopus* embryos were injected with *mRFP* mRNA only (control), or together with Vangl2 or Vangl2-Long MOs, at 4-cell stage in the two dorsal blastomeres, and grown until stage 16. Embryos were immunostained with mAb 36E3 and anti-ZO1. Note the reduction of 36E3 IF signals in Vangl2 or Vangl2-long morphant cells, revealed by the mRFP injection tracer (red). In all panels, scale bars represent 10µm. (**D’**) The intensity of 36E3 immunofluorescent signals was measured with ImageJ/FIJI in uninjected and injected cells, from control and morphant embryos, and plotted on a graph, as indicated. Note that both Vangl2 and Vangl2-Long MO2 significantly reduced 36E3 IF signal intensity. Statistical analyses were done with GraphPad Prism software with a Student’s *t*-test to evaluate significance.

**Figure S6. Rescue of ciliogenesis in Vangl2 and Vangl2-Long morphants.** **(A)** Histogram showing the class distribution of embryos injected as indicated. The bars represent the proportions of embryos in each class calculated for 5 independent experiments. The total number of embryos analyzed for each condition is given above each bar. Vangl2-Long MO1 and Vangl2-Long MO2 caused morphogenetic defects of comparable severity. **(B)** Spatial distribution of Vangl2 proteins revealed by immunofluorescence with the 36E3 mAb in the epidermis at stage 20. Co-staining with the cilia marker Acetylated-Tubulin (Ac-Tub) reveals an enrichment of Vangl2 proteins in MCCs. In all panels, scale bars represent 10µm. **(C)** Embryos were injected at the 4-cell stage into ventral blastomeres with control, Vangl2 MO or Vangl2-Long MO2 morpholinos, and mRFP mRNA, as tracer, as indicated. The same embryos were injected at the 8-cell stage with mRNAs encoding GFP-Vangl2 or GFP-Vangl2-Long. Cells that received both RFP and GFP constructs appeared yellow. Note that Vangl2 and Vangl2-Long MO2 impaired cilia formation, revealed by Ac-Tub immunostaining, which was rescued by both constructs. In all panels, scale bars represent 10µm. **(C’)** The graph represents the percentage of injected MCCs that display normal or abnormal ciliogenesis in all conditions.

**Figure S7. All Vangl2 variants are phosphorylated in *Xenopus* embryos.** *Xenopus* embryos extracts were subjected to Lambda Phosphatase treatment, after immunoprecipitation with mAb 36E3, followed by a Western blot analysis of Vangl2 isoforms using mAb 36E3.

**Table S1. List of Vangl2 peptides detected by L-C MS/MS in the material immunoprecipitated from *Xenopus* embryos with the VANGL2-specific mAb 36E3 antibody**. (1) Vangl2 A and B are two isoforms encoded by the *Xenopus laevis* *Vangl2* loci, *Vangl2-L* and *Vangl2-S*, respectively; (2) MH+ correspond to the monoisotopic mass of monocharged peptide; (3) PSM: total number of identified peptide sequences (peptide spectrum matches) for the protein, including those redundantly identified; (4) PEP: posterior error probability is the probability that the observed PSM is incorrect.

**Table S2. LC-MS/MS mass spectrometry analysis of proteins co-immunoprecipitating with xVangl2** (1) TOP3: calculated as the mean of the three highest peptides areas measured for each protein; (2) #peptides: number of peptide sequences unique to a protein group. (3) PSM: total number of identified peptide sequences (peptide spectrum matches) for the protein, including those redundantly identified; (4) Vangl2 A and B are two isoforms encoded by the *Xenopus laevis* *Vangl2* loci *Vangl2-L* and *Vangl2-S,* respectively.

**Table S3: list of plasmid constructs used in this study**
